# Multiple sulfur isotope signatures of sulfite and thiosulfate reduction by the model dissimilatory sulfate-reducer, *Desulfovibrio alaskensis* str. G20

**DOI:** 10.3389/fmicb.2014.00591

**Published:** 2014-11-25

**Authors:** William D. Leavitt, Renata Cummins, Marian L. Schmidt, Min S. Sim, Shuhei Ono, Alexander S. Bradley, David T. Johnston

**Affiliations:** ^1^Department of Earth and Planetary Sciences, Harvard UniversityCambridge, MA, USA; ^2^Department of Earth and Planetary Sciences, Washington University in St. LouisSt. Louis, MO, USA; ^3^Department of Ecology and Evolutionary Biology, University of MichiganAnn Arbor, MI, USA; ^4^Department of Earth, Atmosphere and Planetary Science, Massachusetts Institute of TechnologyCambridge, MA, USA; ^5^Division of Geological Sciences, California Institute of TechnologyPasadena, CA, USA

**Keywords:** microbial sulfate reduction, multiple sulfur isotopes, biogeochemical sulfur cycle, thionates, sulfur intermediates

## Abstract

Dissimilatory sulfate reduction serves as a key metabolic carbon remineralization process in anoxic marine environments. Sulfate reducing microorganisms can impart a wide range in mass-dependent sulfur isotopic fractionation. As such, the presence and relative activity of these organisms is identifiable from geological materials. By extension, sulfur isotope records are used to infer the redox balance of marine sedimentary environments, and the oxidation state of Earth's oceans and atmosphere. However, recent work suggests that our understanding of microbial sulfate reduction (MSRs) may be missing complexity associated with the presence and role of key chemical intermediates in the reductive process. This study provides a test of proposed metabolic models of sulfate reduction by growing an axenic culture of the well-studied MSRs, *Desulfovibrio alaskensis* strain G20, under electron donor limited conditions on the terminal electron acceptors sulfate, sulfite or thiosulfate, and tracking the multiple S isotopic consequences of each condition set. The dissimilatory reduction of thiosulfate and sulfite produce unique minor isotope effects, as compared to the reduction of sulfate. Further, these experiments reveal a complex biochemistry associated with sulfite reduction. That is, under high sulfite concentrations, sulfur is shuttled to an intermediate pool of thiosulfate. Site-specific isotope fractionation (within thiosulfate) is very large (^34^ε ~ 30‰) while terminal product sulfide carries only a small fractionation from the initial sulfite (^34^ε < 10‰): a signature similar in magnitude to sulfate and thiosulfate reduction. Together these findings show that microbial sulfate reduction (MSR) is highly sensitive to the concentration of environmentally important sulfur-cycle intermediates (sulfite and thiosulfate), especially when thiosulfate and the large site-specific isotope effects are involved.

## Introduction

The geological record preserves only select snapshots of paleo-environments. One of the more robust continuous records of paleo-redox is stored in sedimentary sulfide and sulfate minerals (Thode et al., 1961; Holland, 1973; Strauss, 1997, 1999; Canfield and Raiswell, 1999; Canfield, 2004; Alroy et al., 2008). The isotopic composition of these phases may serve as a prominent proxy for the oxidation state of Earth surface environments (Berner and Canfield, 1989; Kurtz et al., 2003; Hayes and Waldbauer, 2006; Halevy et al., 2012). For instance, the partial pressure of oxygen in Earth's atmosphere is thought to control concentrations of dissolved sulfate and oxygen in the oceans, which in turn may be recorded by the difference in the isotopic compositions between sulfate and sulfide minerals (Canfield, 2001a,b; Habicht and Canfield, 2001; Habicht et al., 2002). At the core of these interpretations is an understanding of the isotope fractionations associated with the numerous redox reactions that characterize the modern sulfur cycle. Among the biologically mediated of sulfur redox reactions is MSR, coupling the oxidation of organic matter or hydrogen to the reduction of sulfate (Peck, 1959, 1962; Rabus et al., 2006; Bradley et al., 2011). MSR is responsible for a large proportion of the organic matter remineralization in anoxic environments (Jorgensen, 1982; Bowles et al., 2014), making it a key environmental process and an important link between the cycles of sulfur carbon and oxygen. MSR is also capable of producing a wide range of mass-dependent sulfur isotope fractionations (Canfield et al., 2010; Johnston, 2011; Sim et al., 2011a; Leavitt et al., 2013). In order to interpret the sulfur isotope variability within geological records—and as it relates to environmental conditions—we first need to understand the controls on the fractionation of sulfur isotopes within the MSR pathway.

**Figure 1:**
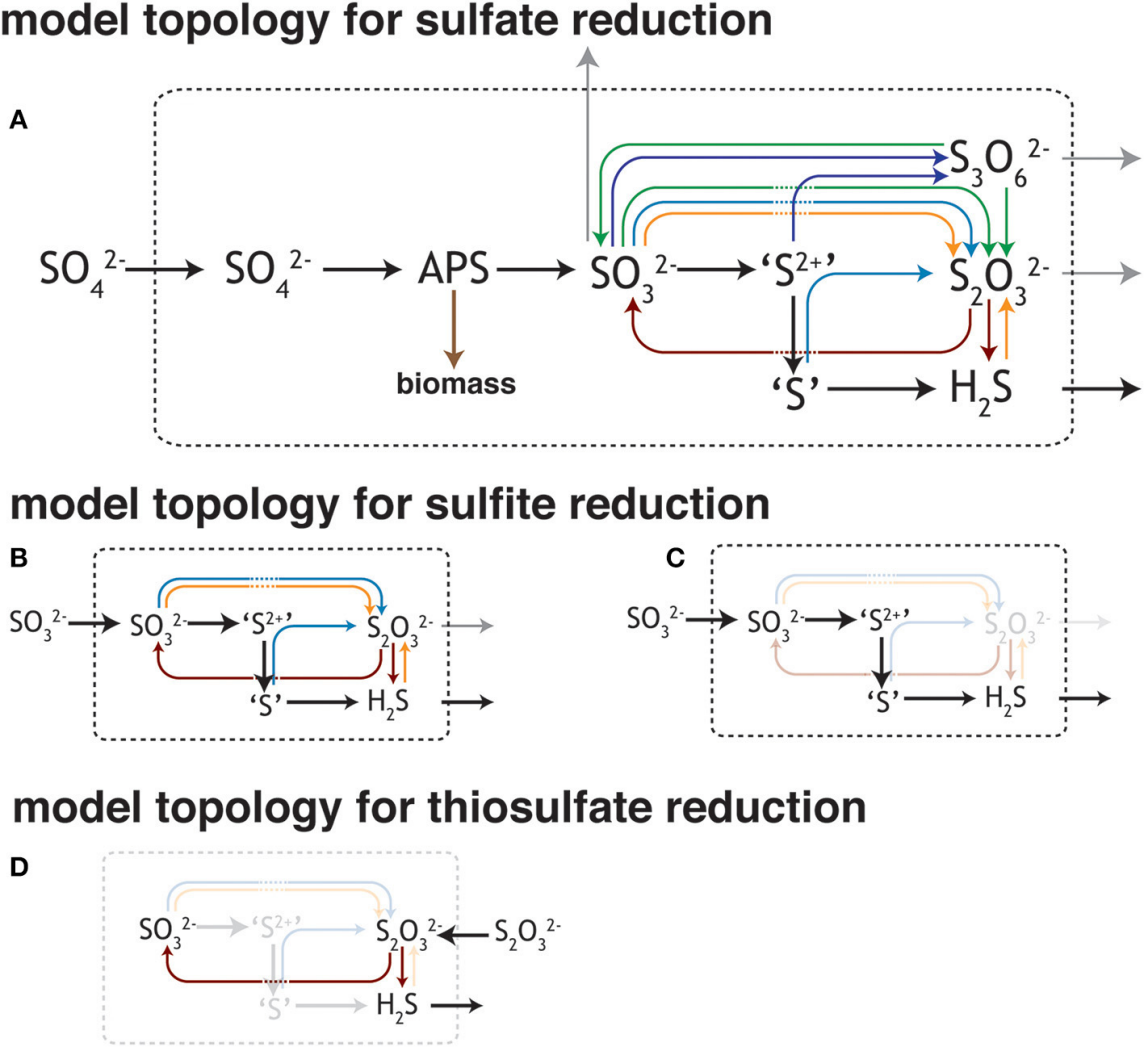
**Different views of the sulfate reduction metabolic network**. **(A)** The model of dissimilatory sulfate reduction modified from Bradley et al. (2011). As it relates to this study, the schematic outlines the potential for numerous reactions between sulfite/bisulfite (HSO^−^_3_/SO^2−^_3_) and hydrogen sulfides/bisulfide (H_2_S/HS^−^) (insets **B–D**). As the isotopic consequences of most of these potential reactions are unknown or under-constrained, this work aims to better assay both the isotope effects and conditions that favor the production of some of these intermediates. Outlined here are the potential reactions for **(B,C)** sulfite and **(D)** thiosulfate [(S-SO_3_)^2−^] reduction, given the standard model of the biochemistry associated with sulfate (SO^2−^_4_) reduction **(A)**. Trithionate [(_3_OS-S-SO_3_)^2−^] is included here, however not observed in this study. This is not an exhaustive depiction of the MSR network, though is a testable topological prediction that would be better informed with future biochemical inquiry (c.f. Venceslau et al., 2014). The valence of the S as it moves through the pathway: sulfate and APS (6+), sulfite (4+), outer S's in trithionate (4+), central S in trithionate (2+), sulfonate S in thiosulfate (5+), reduced S in thiosulfate (0 to 1−), sulfide/hydrogen sulfide (2−). The values for trithionate are best estimates, while those for thiosulfate are from the literature (Vairavamurthy et al., 1993).

Studies of the sulfate reduction metabolism often converge on the idea that sulfate and electron donor availability control the rates of reduction, and in turn, the expressed isotopic fractionation (Harrison and Thode, 1958; Kaplan and Rittenberg, 1964; Chambers et al., 1975; Goldhaber and Kaplan, 1975; Canfield, 2001b; Habicht et al., 2002, 2005; Bradley et al., 2011; Sim et al., 2012; Leavitt et al., 2013). Simply, these variables determine the capacity to deliver reductant to the respiratory reaction network and the relative rates at which electrons and S-bearing oxidants are supplied to catabolic enzymes. The isotopic fractionation associated with MSR is visualized through a schematic depiction (Figure 1A) of the central metabolism (Rees, 1973; Brunner and Bernasconi, 2005; Bradley et al., 2011). This schematic has evolved as our understanding of the metabolism has become more biochemically informed, beginning with a simple three step process (Harrison and Thode, 1958), through to a revised reaction series with the first thorough mathematic derivation (Rees, 1973), to one taking on a more involved reaction chain (Brunner and Bernasconi, 2005), and finally to the most recent update that incorporates a variety of biochemical and enzyme structural information (see Figure 1; Bradley et al., 2011), only available in the last few years (Oliveira et al., 2008, 2011; Venceslau et al., 2010). Much is gained through a close reading of the MSR network as presented in Figure 1. For example, the fractionations classically prescribed to this pathway are associated with sulfate uptake by the cell, the reduction of activated sulfate (APS: adenosine 5′-phosphosulfate) to sulfite, and the terminal reduction of sulfite to sulfide. Given older empirical limits from lab experiments, the maximum fractionation capacity was inferred as the sum of these three steps (with fractionation factors of +3, −25 and −25‰, respectively and 47‰ in total) (Harrison and Thode, 1958; Kaplan and Rittenberg, 1964; Rees, 1973; Chambers et al., 1975). However, recent work with pure and enrichment cultures demonstrated an increased fractionation capacity (up to 66‰) of MSR (Canfield et al., 2010; Sim et al., 2011a; Leavitt et al., 2013), approaching theoretical predictions (~74‰) from low temperature equilibrium exchange (Farquhar et al., 2003; Johnston et al., 2007). These observations require a careful reevaluation of isotopic fractionation and path sulfur follows during MSR.

Determining fractionation factors within the MSR metabolism necessitates a multi-faceted approach. Classic isotope theory states that the kinetic isotope effects of a particular step are only expressed if that step is rate limiting within the reaction scheme (Hayes, 2001). Each reaction that outpaces the rate-limiting step will quantitatively transform its particular substrate to its product, precluding any isotopic discrimination. In reality, reaction chains (particularly in biological systems) do not behave so simply, as multiple steps may compete for rate limitation. The expressed fractionation associated with a particular reaction may instead be a function of how material is transported through a system (e.g., a linear vs. branched/vectorial pathway, Johnston et al., 2007; Bradley et al., 2011). Given these considerations, the network topology in Figure 1 provides a roadmap for identifying the significant reactions within MSR, highlighting where experimental work is most needed. As in Figure 1, sulfite availability serves as a major factor in determining both the reversibility of the MSR network and whether intermediates (such as thiosulfate) factor into the reaction scheme. Fortunately, the physiology of most cultured sulfate reducers carries some plasticity, allowing them to utilize MSR intermediates as terminal electron acceptors in place of sulfate. For instance, *Desulfovibrio alaskensis* strain G20 (G20) will reduce sulfite (SO^2−^_3_) or thiosulfate (S_2_O^2−^_3_) in lieu of or in addition to sulfate (SO^2−^_4_) (Price et al., 2014). Here we present and discuss the results from closed-system (batch) experiments with an axenic culture of G20. For each experiment we account for sulfur isotope mass balance and project the major and minor S-isotope fractionation factors during the dissimilatory reduction of three different electron acceptors: sulfate, thiosulfate, and sulfite. Further, we compare these empirically derived values to calculations of the equilibrium fractionations between the relevant sulfur species. These data shed new light on the inner workings of the sulfate reduction metabolism, illustrate the range of isotopic potential intrinsic to these reactions, and capture a complex chemistry that blurs the lines between the classic picture of MSR and sulfur disproportionation reactions.

The experiments in this study were designed to test our current understanding of how MSR behaves under conditions where the reaction intermediates sulfite or thiosulfate are available at high concentrations. This study tested the biochemistry, physiology, and isotopic fractionations associated with the MSR reaction network (Figure 1). In the classic models for MSR the sulfate anion is imported into the cell, where it can be either exported from the cell in a “back reaction” or activated to APS at the expense of cellular energy reserves (ATP; Peck, 1959). The fate of APS is similarly bidirectional, with both intracellular sulfate and sulfite as possible products (Peck, 1962), where the reduction of APS requires a two-electron gain and the oxidation a two-electron loss. Once as sulfite, there exist numerous possible reaction pathways (Bradley et al., 2011), some of which are directly tested in this study (see Results and Discussion).

The aim of this study was to determine the multiple sulfur isotope fractionation factors (^33^α, ^34^α) between reactant and product, during dissimilatory thiosulfate or sulfite reduction, as compared to control dissimilatory sulfate reduction experiments. This study was conducted with axenic cultures of *D. alaskensis* strain G20, and illustrates the metabolic potential of the MSR network laid out in Figure 1. We confirm growth with sulfate, sulfite and thiosulfate as the sole provided electron acceptors. In keeping with the thermodynamic treatment and recent observations in similar metabolic systems (Shirodkar et al., 2011), the presence of certain sulfoxy anions (e.g., thiosulfate) does not necessitate their reduction if a more energetically favorable electron acceptor is available (e.g., sulfite). Data and Discussion in support of this follow.

## Methods

Experiments were conducted to determine the isotopic fractionation between electron acceptor (sulfate, sulfite or thiosulfate) and product sulfide during closed-system growth of *D. alaskensis* on lactate (electron donor). In addition, sulfate reduction experiments were conducted with either lactate or formate as the sole provided electron donor, whereas sulfite and thiosulfate reduction experiments were paired with lactate only. A pure culture of the sulfate-reducing bacterium *D. alaskensis* strain G20 was grown in airtight glass Balch tubes sealed with butyl rubber septa under a gas headspace of 90% nitrogen and 10% carbon dioxide. Media was degassed with this mix prior to inoculation. The medium consisted of (per liter) NaCl, 20 g; MgCl_2_·6H_2_O, 3 g; CaCl_2_·2H_2_O, 0.15 g; NH_4_Cl, 0.25 g; KH_2_PO_4_, 0.2 g; KCl, 0.5 g; Na_2_SeO_4_·10H_2_O, 370 mg, as well as a vitamins and amino acids solution and a trace metals solution (Widdel and Bak, 1992). Electron acceptors were provided at 20 mM (sulfite or sulfate) or 10 mM (thiosulfate), initial concentrations), while electron donor was always provided at 10 mM (lactate or formate). All medium was prepared after solutions were degassed with O_2_-free N_2_ for at least 1 h/L, and transferred in an anaerobic chamber under an atmosphere of N_2_:H_2_ 95:5. In each experiment un-inoculated controls were monitored for contamination, and a killed control to quantify any inoculum sulfur. All cultures were grown in their respective media for >10 transfers prior to the inoculation of the experiments reported here. Bacteria were transferred at a 1:100 dilution to guarantee the quantity of reduced sulfur carried over with the inoculum was below 10 mM. Each tube was sampled at roughly five time points spread throughout each experiment to ensure capture of exponential phase growth.

At each sampling time (*t*), including at the start of the experiment (*t* = 0), optical density and chemistry measurements were performed. The optical density of each tube was measured at A600. These measurements were calibrated to absolute cell counts through a standard staining protocol (Moore et al., 1998). In parallel, concentrations of relevant chemical species were measured throughout. Sulfide was quantified colorimetrically (Cline, 1969) (detection limit of 50 μM, given the protocol used herein) whereas sulfate, thiosulfate and sulfite were all measured via ion chromatography (Leavitt, 2014) (detection limit 10 μM). Given the instability of sulfite under ambient atmosphere (O_2_), care was taken to avoid oxidation by preserving samples with formalin (0.1 mL of 600 mM anoxic formaldehyde added to each 1 mL of sample) (Leavitt, 2014). Sulfate concentrations were measured using an isocratic method, while sulfite and thiosulfate were measured using gradient elution. Trithionate was independently measured by cyanolysis (Kelly and Wood, 1994), though was never detectable (detection limit 40 μM). More sensitive methods for detecting trithionate or thiosulfate do exist, though were not employed in this study (Newton and Fahey, 1995). Elemental sulfur was detected as chromium reducible sulfide (CRS) (Canfield and Desmarais, 1994). Sulfur species were separated and prepared for major and minor isotope determination methods by established protocols (Leavitt et al., 2013; Leavitt, 2014).

Sulfur isotope measurements of sulfate, sulfide and thiosulfate sulfur were performed first on a Thermo-Finnegan Delta V mass spectrometer, configured in continuous flow mode and connected to an Elemental Analyzer (measuring SO_2_). Given the above chemical methods, sulfate and sulfonate were measured as BaSO_4_ whereas sulfide and reduced thiosulfate S were measured as Ag_2_S (data are reported in Tables 1–3), always with an excess of V_2_O_5_. From this, select data were chosen for high precision analyses via dual inlet on a Thermo Finnigan MAT 253 (as SF^+^_5_). Samples already as silver sulfide were fluorinated directly with an excess of pure F_2_, cleaned cryogenically and via gas chromatography before introduction to the mass spectrometer. Samples as BaSO_4_ precipitates were chemically reduced to Ag_2_S (Forrest and Newman, 1977) prior to high precision analyses.

Table 1–4**All geochemical, microbial, and isotopic data from batch experiments**.

**Table:** 

**Table 1**		***n* (replicates)**	**Time (hours)**	**Cells (10^6^/mL)**	**RSD (%)**	csSRR (*f*mol/cell*day)	***f* (%)**	**[SO_4_] (mM)**	**σ_[SO4]_ (mM)**	**[HS^−^] (mM)**	**σ_[HS-]_ (mM)**	**^34^ε (‰)**
Sulfate	Lactate	3	0	3.4	5.8	0.0	0.0	20.24	2.33	0.01	0.00	0.00
		3	24	12.2	25.6	12.8	2.2	20.19	1.03	0.45	0.15	−4.02
		3	29.4	71.6	35.2	142.3	6.9	19.24	0.21	1.39	0.43	−4.25
		3	34.1	309.1	10.1	80.7	16.2	17.36	0.15	3.28	0.29	−3.87
		3	39.5	423.9	1.3	61.8	18.9	16.56	0.15	3.82	0.12	−4.86
		1	n.c.	6.0				20.06		0.00		
		1	k.c.	6.2				24.51		0.01		
Sulfate	Formate	3	0	3.07	5.89	0.00	0.00	18.00	0.00	0.00		
		3	69	6.52	12.76	494.48	10.28	15.55	0.94	1.85	0.30	−6.55
		3	81	17.69	34.16	420.70	21.80	14.38	1.33	3.92	0.54	−5.34
		3	104.5	32.21	5.47	134.19	28.08	13.42	0.08	5.05	0.15	−5.88
		1	n.c.	64.18				19.06		0.01		
		1	k.c.	6.20				18.20		0.02

**Table:** 

**Table 2**		***n* (replicates)**	**Time (hours)**	**Cells (10^6^/mL)**	**RSD (%)**	csSRR (*f*mol/cell*day)	***f* (%)**	**[S_2_O_3_] (mM)**	**σ_[S2O3]_ (mM)**	**[HS^−^] (mM)**	**σ_[HS-]_ (mM)**	δ^34^S_[S* O3]_ (‰)	δ^34^S_[*S]_ (‰)	**δ^34^S_[HS-]_ (‰)**
Thiosulfate	Lactate	3	0	1.54	0.56	0.00	0.00	18.00	0.07	0.00	0.00	2.16	−2.16	0.00
		3	13	21.46	0.47	134.91	2.02	17.96	0.29	0.73	0.07	0.03	−2.81	−8.61
		3	19	71.45	0.27	264.91	6.62	16.87	0.28	2.38	0.15	0.64	−2.61	−8.32
		3	33	138.99	0.20	67.25	10.30	16.11	0.04	3.71	0.12	3.42	−1.26	−6.67
		3	40	300.46	0.17	66.09	14.62	13.80	0.00	5.26	0.21	4.34	−1.46	−6.02
		1	n.c.	0.06	0.00			17.53						
		1	k.c.	3.20	0.02			17.76

**Table:** 

**Table 3**		***n* (replicates)**	**Time (hours)**	**Cells (10^**6**^/mL)**	**RSD (%)**	csSRR (*f*mol/cell*day)	***f_**H2S**_* (%)**	***f_**S2O3**_* (%)**	**[SO_**3**]_ (mM)**	**σ_[SO4]_ (mM)**	**[HS^−^] (mM)**	**σ_[HS-]_ (mM)**	**[S_**2**_O_**3**]_ (mM)**	**σ_[S2O3]_ (mM)**	**δ^**34**^S_[SO3]_ (‰)**	**δ^**34**^S_[HS-]_ (‰)**	δ^**34**^S_[S* O3]_ (‰)	δ^**34**^S_[* S]_ (‰)
Sulfite	Lactate	3	0	2.53	26.36	0.00	0.00	0.00	17.11	0.50	0.06	0.02	0.04	0.00	0.00	0.00	0.00	0.00
		3	20	36.55	9.59	141.65	1.99	8.56	15.10	0.15	0.34	0.02	0.73	0.09	0.13	−10.27	0.00	0.00
		3	23.4	108.29	5.14	452.96	4.91	21.47	12.80	0.08	0.84	0.07	1.84	0.14	0.41	−6.14	16.78	−15.19
		3	26.5	227.33	3.58	408.33	9.70	37.20	9.66	0.16	1.66	0.06	3.18	0.12	1.29	−3.16	15.38	−13.62
		3	30.6	469.77	0.30	180.89	15.55	52.12	5.92	0.60	2.66	0.20	4.46	0.82	0.61	−2.54	14.16	−13.29
		1	n.c.	1.56					18.23		0.02		0.04					
		1	k.c.	0.20					16.72		0.02		0.04

**Table:** 

**Table 4**		**Sulfate**		**Sulfite**		**Sulfide**		**Sulfonate**		**Reduced S**		**Elemental sulfur**	
		**δ^**34**^S**	**Δ^**33**^S**	**δ^**34**^S**	**Δ^**33**^S**	**δ^**34**^S**	**Δ^**33**^S**	**δ^**34**^S**	**Δ^**33**^S**	**δ^**34**^S**	**Δ^**33**^S**	**δ^**34**^S**	**Δ^**33**^S**
Sulfate	Lactate	0.27	−0.02	−	−	−3.95	0.008	−	−	−	−	−	−
Sulfate	Formate	1.76	−0.01	−	−	−5.15	0.016	−	−	−	−	−	−
Sulfite	Lactate	−	−	1.29	0.033	−3.16	0.012	15.38	0.023	−13.62	0.002	−	−
		−	−	0.61	0.038	−2.54	0.022	14.16	0.041	−13.29	0.041	−	−
Thiosulfate	Lactate	−	−	−	−	−	−	2.16	−0.037	−2.16	0.039	−	−
		−	−	−	−	−6.67	0.013	3.42	−0.017	−1.26	0.006	−5.58	0.019
		−	−	−	−	−6.02	0.024	4.34	−0.005	−1.46	0.009	−	−

For the two sulfate (Table 1) experiments, the corrected fractionation factors are presented (^34^ε). For thiosulfate and sulfite experiments (Tables 2, 3), where unique fractionations between product and reactant can be calculated, values are noted as (δ^34^S are reported, ^34^ε are calculated in the text), whereas when values are more difficult to ascertain, the direct isotopic compositions of the measured pools are reported. See text for full Discussion and equations for each. Note that reported thiosulfate concentrations are for total sulfur. Minor isotope data is presented in Table 4 for the time point(s) characterized, all relative to VCDT.

All isotope data presented herein is in standard delta notation, where the composition of a given sample is normalized (in our case) back to the original composition of the sulfoxy anion in the experiment. In the case of thiosulfate experiments, samples are normalized to the composition of the bulk S_2_O^2−^_3_. This results in two distinct delta values—δ^33^*S* and δ^34^*S* (the ratios of ^34^*S*/^32^*S* in a standard relative to that of a reference). Values for δ^34^*S* are from both SO_2_ and SF_6_ measurements, whereas δ^33^*S* data are exclusively from SF_6_ measurements. When the composition of two reservoirs is being related, we define ^34^ε [(^34^α − 1) × 1000] and where ^34^α between reservoirs A and B is (δ^34^*S*_*A*_/1000 + 1)/(*d*^34^*S*_*B*_/1000 + 1). The triple isotope composition of a given reservoir can be related through:

$$\Delta^{33}S=\delta^{33}S-1000\times \left[\left(1+{\left(\delta^{34}S/1000\right)}^{0.515}\right)-1\right].$$

When two pools are being related in triple isotope space, we can use the slope of the line on a δ^33^S vs. δ^34^*S* plot, or:

$${}^{33}\lambda =ln{(}^{33}\alpha )/ln{(}^{34}\alpha ).$$

For further information about the meaning and specific controls on mass-dependent fractionations, readers are referred to published discussions (Miller, 2002; Young et al., 2002; Farquhar and Wing, 2003; Johnston et al., 2005; Johnston, 2011).

To calculate the fractionation factor of a given reaction, we apply a standard closed-system Rayleigh model with respect to the reactant:

(1) $$\delta^{34}S_{R,t}=[((\delta^{34}S_{R,\;t\;=\;0})+1000)\times f_{t}^{\left(3x_{\alpha -1}\right)}]-1000.$$

where δ^34^*S*_*R*, *t*_ is the isotopic composition (*x* = 3 or 4) of the reactant (*R*) though time (0, *t*). For this expression, the reaction coordinate is tracked with *f*, which represents the mole fraction of reactant remaining at time *t*. Additionally, the composition of a given product is calculated:

(2) $$\begin{array}{l} \delta^{34}S_{P,t}=[\left({\left[R\right]}_{0}\times \delta^{34}S_{R,t\;=\;0}\right)-\left(f_{t}\times \delta^{34}S_{R,t}\right)\\ \;+\left({\left[P\right]}_{0}\times \delta^{34}S_{P,t\;=\;0}\right)]/{\left[R\right]}_{t}. \end{array}$$

where the sulfur isotope composition of the reactant (*R*) or product (*P*) are related to their concentrations ([*R*] and [*P*]) at a given time point (*t*) (also see Equation 9 in Ono et al., 2012).

Isotopic equilibrium can be calculated as the reduced partition function ratios of the different isotopologues (Bigeleisen and Mayer, 1947; Urey, 1947). For partition function calculations, the necessary vibrational frequencies for H_2_S, SO^2−^_3_, S_2_O^2−^_3_, and SO^2−^_4_ were obtained by quantum chemical calculations. Herein we use the Gaussian03 software package to optimize geometry and calculate vibrational frequencies. The Hartree–Fock (HF) method and 6-31G^*^ basis set without symmetry constraints were used for both geometry optimization and frequency calculation. As the HF theory is known to systematically overestimate fundamental frequencies (Scott and Radom, 1996), the scaling factor of 0.8928 was determined using a least-squares approach (Scott and Radom, 1996) and the experimental frequencies for H_2_S (81), and applied to the calculated frequencies. To determine the shifts in vibrational frequencies upon isotopic substitution, the same calculation was made for each isotopologue in which one ^32^*S* atom was replaced by ^34^*S*. For all calculation processes, solution effects were included using the polarizable continuum model (PCM) (Miertus et al., 1981; Miertus and Tomasi, 1982).

## Results and discussion

Four discrete experiments were performed in this study. In each case, the dominant electron acceptor in the system was consumed with time as one or more reduced S-products accumulated. In two experiments G20 was grown on sulfate as the sole terminal electron acceptor and either lactate or formate as the sole electron donor. In the two additional experiments, only lactate was provided as the electron donor with either sulfite or thiosulfate as the sole provided terminal electron acceptor. For each, we quantify the loss of reactants and generation of products to explicitly track elemental and isotopic mass balance at each time point. Given the complex chemistry involved—for sulfite reduction in particular—we emphasize the requirement to explicitly measure each S pool at each time-point to close isotope mass-balance. When coupled with optical densities and cell counts, cell specific reduction rates are also calculated (Detmers et al., 2001).

The nature of the experiments (closed-system) requires additional calculations to determine the fractionation factor (^3*x*^α). Fortunately, our experimental methods circumvented an inoculum sulfide blank, streamlining previous mathematical treatments (Johnston et al., 2007). In the simplest case, the loss of a reactant and generation of a single product pool is then determined through a standard closed-system “Rayleigh” fractionation model (Nakai and Jensen, 1964) (Equations 1 and 2). It is through the use of these two expressions that we define isotope fractionation effects in our experiments.

## Sulfate reduction

**Figure 2:**
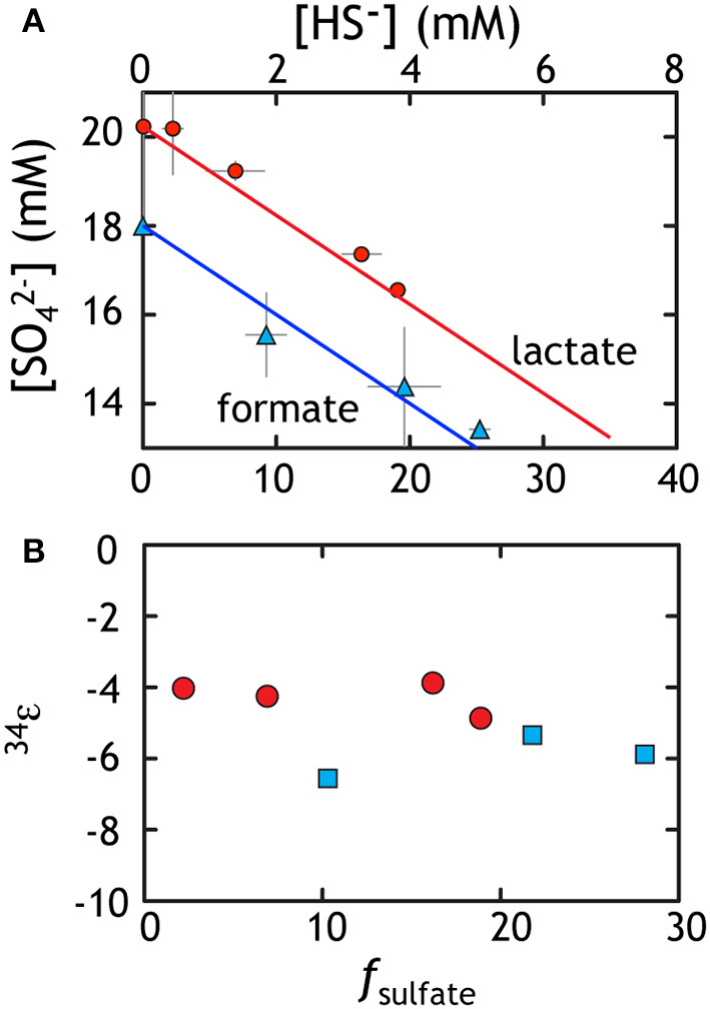
**Data from sulfate reduction experiments with lactate (red) or formate (blue)**. **(A)** The loss of sulfate is accounted for with the ingrowth of sulfide. Data reflect various time points throughout the experiment and the line corresponds to perfect closure of elemental mass balance (theory). **(B)** The calculated fractionation factor (^34^ε) relating sulfate and sulfide using *f* and the equations from the text. The isotopic residuals (to satisfy the calculated mass-imbalance) on the sulfate experiments are 0.18 and 0.42‰ in δ^34^*S* and 0.007 and 0.017‰ in Δ^33^*S* for formate and lactate, respectively.

Sulfate reduction experiments with both lactate and formate generated sulfide as the lone S-bearing product. Near the end of exponential phase growth, 28.1 and 18.9% of the available sulfate had been consumed by formate and lactate oxidation, respectively (Figure 2A). This results in cell specific sulfate reduction rates (csSRRs) during exponential phase ranging up to 142 and 494 *f*mol per cell per day for formate and lactate oxidation, respectively. These rates are calculated from the change in sulfate concentrations between adjacent time points, divided by the difference in average cell count during that interval (Detmers et al., 2001). The same calculation can be performed with the product sulfide concentrations. These results are consistent with csSRR for related strains grown in batch (cf. Harrison and Thode, 1958).

The fractionation associated with sulfate reduction was calculated using Equation (1), described above. For the formate oxidation experiment, the net ^34^ε_*SO*4/*H*2*S*_ averaged −4.25 ± 0.44‰, whereas growth on lactate resulted in slightly larger fractionation of −5.93 ± 0.61‰ (Figure 2B). At higher rates where electron donor and acceptor and not initially limiting, like those here, fractionation approaches a minimum near 4‰ (Harrison and Thode, 1958), perhaps reflecting the fractionation associated with converting sulfate to sulfite. As rates slow, fractionation can range up to >66‰ (Sim et al., 2011a,b; Leavitt et al., 2013), approaching the predicted equilibrium fractionation (Tudge and Thode, 1950; Farquhar et al., 2003; Johnston et al., 2007), presumably by a relaxation of the thermodynamic driving force (Thullner et al., 2008) For the purposes of this study, we take these fractionations as a representative baseline for the growth of G20 under sulfate replete conditions. These data are then directly comparable to growth on the other terminal electron acceptors tested here under similar closed system conditions with initially non-limiting electron donor and acceptor.

These experiments also serve to extend the catalog of minor sulfur isotopic data for sulfate reduction. From earlier works, it is clear that the MSR process is broadly characterized as having highly variable ^34^ε effects with ^33^λ that is exclusively less than equilibrium predictions of 0.515. Minor isotope fractionations range down to less than 0.510 with a mean near 0.512 (Farquhar et al., 2003; Johnston et al., 2005, 2007; Canfield et al., 2010; Bradley et al., 2011; Johnston, 2011; Sim et al., 2011a,b, 2012; Leavitt et al., 2013) showing significant co-variance with csSRR (Sim et al., 2011b; Leavitt et al., 2013) Consistent with these findings, the sulfate reduction experiments from this work yield calculated ^33^λ of 0.508 ± 0.002 and 0.511 ± 0.001 for lactate and formate, respectively (Table 1). It is important to note that the error on ^33^λ is heavily (and non-linearly) dependent on ^34^ε, meaning that at low ^34^ε, the error is larger and the ^33^λ less uniquely resolvable. Here we use updated error propagation equations derived previously (Johnston et al., 2007).

**Figure 3:**
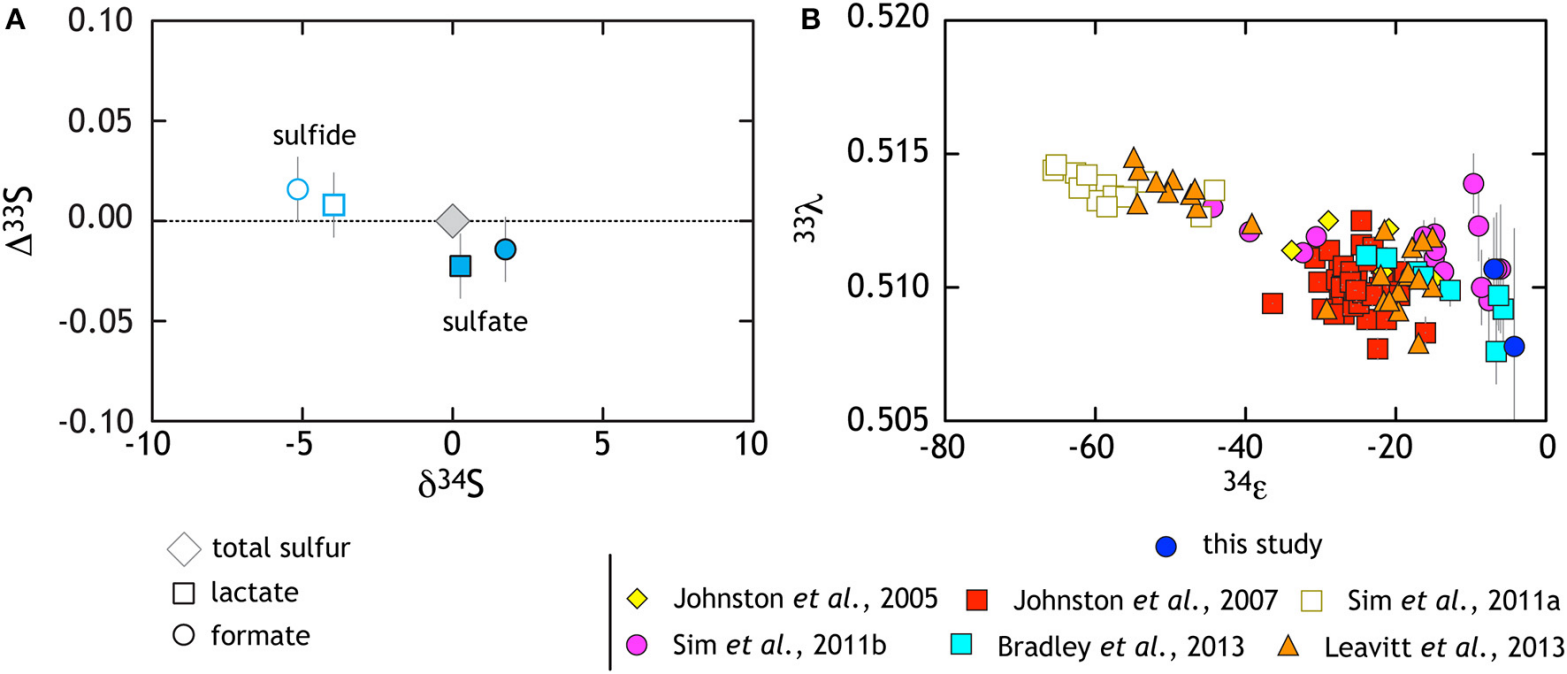
**The triple isotope consequences of sulfate reduction are presented in two complementary frames. (A)** Data from lactate and formate experiments are presented relative to the starting composition (gray diamond). White symbols with colored outlines are sulfide data whereas filled symbols are sulfates (see key for further description). Here, sulfides become characteristically depleted in δ^34^*S* and enriched in Δ^33^*S*, whereas sulfate preserves the opposite behavior. **(B)** Compilation of pure culture sulfate reduction experiments where multiple S isotope values are reported (references in legend).

Along with these new data, we recalculated (i.e., normalized) and compiled published pure culture results from both open- and closed-system sulfate reduction experiments (Figure 3B). The compilation highlights a number of key observations. Foremost among these is the resolvable triple isotope trend where elevated ^33^λ corresponds to larger ^34^ε fractionation, as recently highlighted in both open and closed system studies (Sim et al., 2011b; Leavitt et al., 2013). Despite this trajectory toward low temperature thermodynamic equilibrium, such theoretical values have not yet been observed in a microbial experiment (Canfield et al., 2010; Sim et al., 2011a,b; Ono et al., 2012; Leavitt et al., 2013). In light of this clear triple isotope relationship, it is important to note that this compilation represents a variety of different MSR strains over a range of conditions. For deeper physiological understanding, further continuous culture (open-system) and theoretical work are necessary. Still, a great deal of isotopic behavior is shared among the different experimental approaches and is likely universal to MSRs.

## Thiosulfate reduction

**Figure 4:**
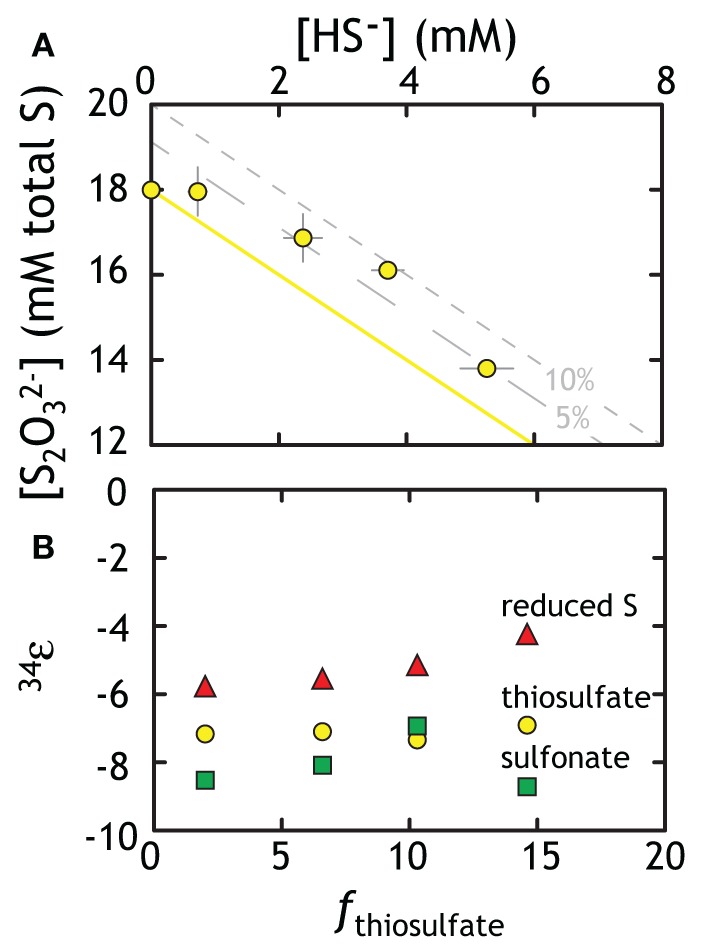
**Geochemical and isotopic data from experiments with G20 as a thiosulfate reducer. (A)** Thiosulfate was lost to the production of sulfide. The yellow line represents ideal closure of mass-balance with the two gray lines representing 5 and 10% excess of total S. **(B)** The three different methods for calculating the fractionation associated with thiosulfate reduction (fractionation based on total thiosulfate, reduced S or sulfonate: see text for Discussion). Put differently, the calculated ^34^ε_X-sulfide_ values describe the intrinsic fractionation factor calculated (using equations in the text) if the precursor S is in different phases. Isotopic data in Table 2 is not subjected to the Rayleigh calculation used to generate Figure 4. The isotopic residuals (calculated mass-imbalance) on the thiosulfate experiments are 0.28 and 0.35‰ in δ^34^*S* and 0.005 and 0.003‰ in Δ^33^*S*.

The thiosulfate reduction experiment with G20 carried straightforward geochemical results, with thiosulfate reduced to hydrogen sulfide (Figure 4A). This is consistent with previous reports for three other strains of sulfate reducers (*D. desulfuricans*, *D. sulfoexigens*, and *D. multivorans*) grown on thiosulfate (Habicht et al., 1998; Smock et al., 1998). Over the course of our experiment, 15% of the thiosulfate sulfur was reduced to sulfide, leading to cell specific thiosulfate reduction rates of up to 265 *f*mol per cell per day during exponential phase (Table 2). However, predictions from earlier work on MSR suggest that sulfite serves as an intermediate between thiosulfate and sulfide (essentially the liberation and subsequent reduction of the sulfonate S) (Smock et al., 1998). Although methods for quantifying sulfite were employed in these experiments, sulfite was never above the detection limit (<10 μM). This means that if present, sulfite has an exceedingly short residence time and is quickly reduced to sulfide. Given this short lifetime, the isotopic consequences of a sulfite intermediate are not likely expressed in this experiment.

Calculating an accurate fractionation factor is more challenging, however, as there are multiple possible reaction pathways to generate sulfide from thiosulfate. There are two unique S sites within a thiosulfate molecule, the potential for intermediates, and the possibility that not only one pathway operates at a time. The three simplest scenarios are (see Figure 1):

(3a) $$S_{2}O_{3}^{2-}\;\to SO_{3}^{2-}+S^{0}\to SO_{3}^{2-}+HS^{-}$$

(3b) $$S_{2}O_{3}^{2-}\;\to SO_{3}^{2-}+S^{0}\to HS^{-}+S^{0}$$

(3c) $$S_{2}O_{3}^{2-}\;\to SO_{3}^{2-}+S^{0}\to 2HS^{-}$$

Beginning with the simplest approach, it is possible that thiosulfate reduction is not site-specific, which is the same as having a wholesale reduction of thiosulfate to bisulfide (8 *e*^−^, Equation 3C). This would generate bisulfide fractionated away from the bulk isotopic composition of thiosulfate. If true, then the fractionation factor is calculated using a typical closed-system “Rayleigh” distillation model, and yields an ^34^ε of −7.12 ± 0.18‰ (Figure 4B). In evaluating the possibility that the oxidized or reduced S in thiosulfate is preferentially reduced (2 and 6 *e*^−^ in Equations 3A,B), we first note a small mass-imbalance (never exceeding 10%) in these experiments (see Figure 4A). We presume that this imbalance relates in part to a small elemental S (S^0^) pool that was measurable from the later time (as chromium reducible sulfur, CRS). This may suggest preferential sulfonate reduction (Equation 3B), leaving the reduced thiosulfate S behind, which contributes to residual S^0^. Using the same mathematical treatment as above, a sulfonate reduction fractionation is calculated as: ^34^ε = −8.06 ± 0.80‰. This solution is statistically indistinguishable (within one standard deviation) from the fractionation factor calculated for wholesale thiosulfate reduction. For completeness, we calculate the predicted fractionation if reduced thiosulfate S reduction alone is responsible for sulfide production (Equation 3A), resulting in an estimate of ^34^ε = −5.17 ± 0.68‰. Given the lack of measurable sulfite, the modest in-growth of S^0^, and the similarity between the sulfonate and full thiosulfate fractionation factors, we again take sulfide production as predominately indiscriminate, wholesale thiosulfate reduction with the potential for modest contributions from preferential sulfonate reduction. Furthermore, the role of intermediates, such as sulfite, remains unclear.

**Figure 5:**
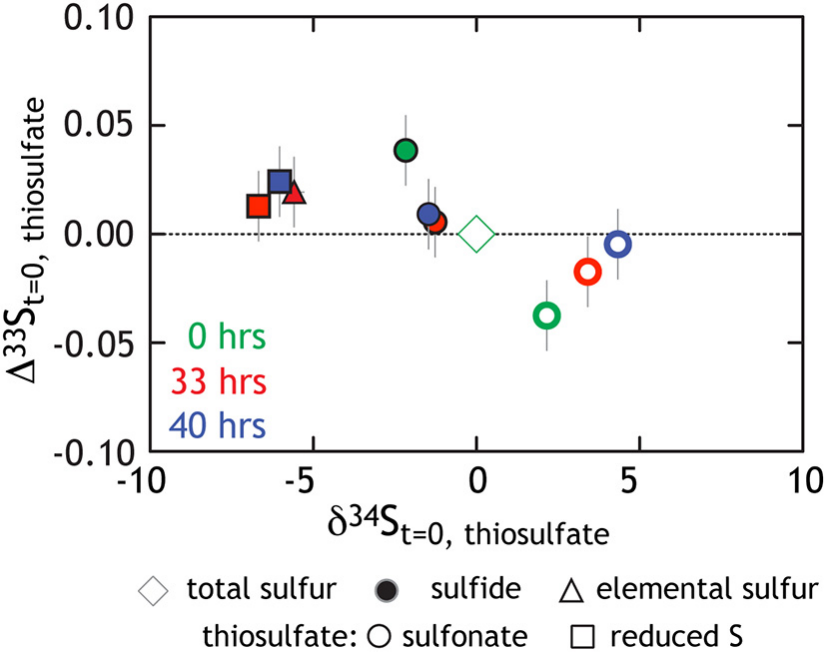
**Triple isotope data for thiosulfate reduction experiments**. As opposed to Figure 4B where we presented calculated ^34^ε values, here all isotope data is normalized to the bulk composition of the starting thiosulfate (green diamond at origin). Also in green are the site-specific compositions of the reduced and sulfonate S (square and circle, respectively). Those same S sites at times 33 h (red) and 40 h (blue) are also included.

This study presents the first minor sulfur isotope data for microbial thiosulfate reduction (Figure 5; Table 4). In a similar fashion to that observed elsewhere (Habicht et al., 1998), the sulfonate S sites become isotopically enriched in ^34^*S* whereas the reduced thiosulfate S becomes more depleted (relative to the net composition of starting thiosulfate). During mid- and late- exponential phase growth (the samples analyzed for ^33^*S*), ^34^*S* enrichment is accompanied by a modest increase in the Δ^33^*S* of sulfonate S and the decrease in Δ^33^*S* of reduced thiosulfate S. In complement to the isotopic evolution of the thiosulfate, product sulfide is isotopically depleted in ^34^*S* and preserves a slightly increased Δ^33^*S*. Under the assumption of wholesale thiosulfate reduction, we can extract a ^33^λ between the bulk composition of thiosulfate and product sulfide of ~0.512 (0.5119 and 0.5124) for both time points presented in Figure 5. This value is remarkably similar to that extracted from sulfate reduction experiments (Figure 3B). This suggests a shared biochemical pathway and/or a commonality in the physical chemistry of both sulfate and thiosulfate reduction by G20. Regardless, the fact that sulfonate and reduced thiosulfate S isotopically balance one another throughout the experiment further suggests wholesale thiosulfate reduction is the pathway, rather than a site-specific reaction.

## Sulfite reduction

The mechanism(s) of sulfite reduction to sulfide are the core of the MSR energy metabolism and the topic of much discussion in both the biochemical and isotope geochemical literature (see Bradley et al., 2011; Venceslau et al., 2014). For this reason, experiments growing sulfate reducers on sulfite are relatively common (Harrison and Thode, 1958; Drake and Akagi, 1978; Fitz and Cypionka, 1989, 1990; Habicht et al., 1998; Smock et al., 1998) and capture two very different manifestations of the MSR reaction network. In the most straightforward experiments, sulfite reduction only produces sulfide (cf. Habicht et al., 1998). Estimated fractionation factors for sulfite reduction suggested a small (~6‰) ^34^ε between residual sulfite and sole product sulfide (Habicht et al., 1998). Conversely, other studies demonstrate the production of other sulfoxy anions (thionates: thiosulfate, trithionate, and tetrathionate) during sulfite reduction that are either intermediate or terminal reaction products (cf. Kobayashi et al., 1969; Fitz and Cypionka, 1990). One such reaction pathway involving intermediates is summarized as three reactions (Akagi et al., 1994):

(4a) $$3SO_{3}^{2-}\;↔\;S_{3}O_{6}^{2-}$$

(4b) $$S_{3}O_{6}^{2-}\;↔\;S_{2}O_{3}^{2-}+SO_{3}^{2-}$$

(4c) $$S_{2}O_{3}^{2-}\;↔\;HS^{-}+SO_{3}^{2-}$$

and results in the net stoichiometry of *SO*^2−^_3_ → *HS*^−^. In this case, trithionate is always the first intermediate produced (Kobayashi et al., 1969; Fitz and Cypionka, 1990), and during the step-wise reduction, sulfite is continually regenerated (Fitz and Cypionka, 1989). It is presumed (and testable) that the sulfonate S is simply shuttled between sulfoxy anions and would, as a result, not change in isotopic composition. If true, the only isotopic consequences would be the result of sulfide production: a consequence isotopically distributed within the sulfite pool. What remains unclear is the relative rates of these reactions, whether they are truly linked and what shared (or unique) enzymes are responsible for catalysis.

This cascade of reactions has been investigated in vitro (Nakatsuk and Akagi, 1969; Haschke and Campbell, 1971; Hatchikian, 1975), *in vivo* with the tracking of electron acceptors and donors (Sass et al., 1992), as well as work interrogating the subcellular localization of the enzymes involved in the reduction (Drake and Akagi, 1978; Venceslau et al., 2010). Despite the simplified overall stoichiometry and general reaction outline (Figure 1), the potential for numerous unique pathways—each with differing reaction rates and residence times in each intermediate phase—could carry significant isotopic consequences. This is all in an attempt to locate the machinery and drivers behind thionate production, and in the end, better understand the reduction of sulfite to sulfide.

**Figure 6:**
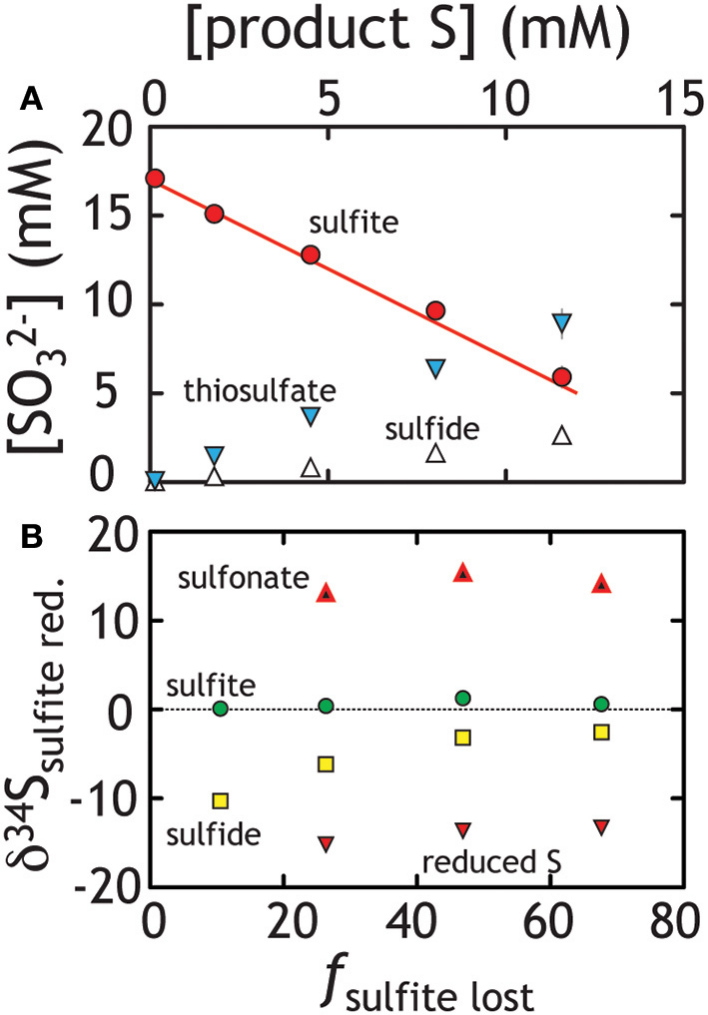
**Sulfite reduction experiments, where both thiosulfate and sulfide were produced**. **(A)** The line represents closure of mass balance (the overlap of the red dots and line). **(B)** The isotopic compositions of the various products and residual reactants over the course of the experiment are presented. Unlike the sulfate and thiosulfate reduction experiments (Figures 2, 4), simple values cannot be calculated uniquely. As such, we present the measured isotopic composition of various pools (δ^34^*S*) relative to the total S in the system rather than a derived ^34^ε value. See text for Discussion.

In these experiments strain G20 grew on sulfite while producing a mixture of products. Rather than simply producing sulfide, G20 follows a stoichiometry similar to Equation 4, with both thiosulfate and sulfide produced in a 2:1 stoichiometry (Figure 6, Table 1). Notably, trithionate was below detection (40 μM) for each experiment. Sulfite loss in paralleled by sulfide and thiosulfate generation at rates approaching 100 and 250 *f*mol per cell per day, respectively (Table 3). These rates are slightly lower than that for sulfate and thiosulfate reduction experiments. The isotopic behavior in these experiments is similarly complex (Figure 6B). The residual sulfite pool only changes composition slightly, becoming more enriched. Product sulfide is initially isotopically depleted relative to residual reactant sulfite (by up to 10‰ in ^34^*S*), whereas sulfonate and reduced thiosulfate S carry much more enriched and depleted values, respectively. Diverging from the prediction of Equation 4, thiosulfate here is produced in excess of sulfide (a 2:1 rather than 1:1 concentration ratio, see Figure 6A). Sulfide and thiosulfate concentrations should change in concert if the only loss mechanism for thiosulfate is to sulfide and sulfite according to Equation 4.

**Figure 7:**
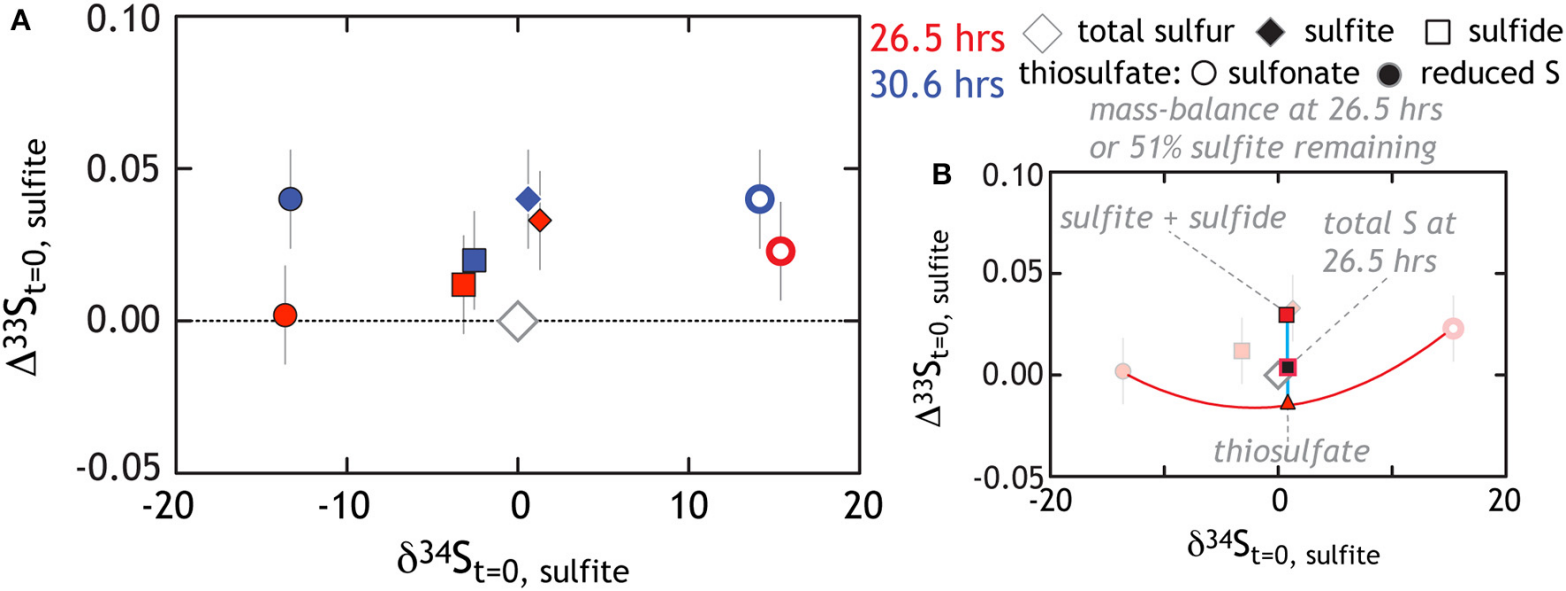
**The triple isotope consequences for sulfite reduction by G20**. All data is plotted relative to the total sulfur at the initiation of the experiment. **(A)** As the experiment progresses sulfite gains a modest ^34^ε and Δ^33^*S* enrichment, while sulfide tracks toward depleted δ^34^*S* with an enriched Δ^33^*S*. Thiosulfate is also generated with reduced and sulfonate falling compositionally very far outside the residual sulfite and product sulfide (see text for further Discussion). **(B)** We use the 26.5 h data (fraction of sulfite remaining is 51%) to demonstrate the degree to which mass-balance is closed. For example, the red triangle is the “total thiosulfate” composition, which occurs at the 50% point of a mixing line between reduced and sulfonate sulfurs. Note that in this coordinate system, mixing is non-linear (Johnston, 2011). Similarly, the red square is the mixture of sulfite and sulfide. The black-red square is the calculated total S at 26.5 h. This value is then directly comparable to the total S at the initiation of the experiment (gray diamond). The isotopic residual (calculated mass-imbalance) on the sulfite reduction experiments are 0.7 and 0.02‰ in δ^34^*S* and 0.013 and 0.024‰ in Δ^33^*S* at 26.5 and 30 h, respectively.

At first pass, we may posit that thiosulfate reduction is the rate limiting reaction. If true, we would predict that the isotopic signature of thiosulfate reduction would be recorded in sulfide and reflect the signature observed in our thiosulfate reduction experiment. Alternatively, thiosulfate production may suggest numerous reactions are occurring in parallel. De-convolving the overall reaction sequence may be aided by multiple S isotope measurements. Despite this complex mixture of products, isotopic mass balance closes as the reaction proceeds (Figures 6A, 7B)^1^. In these experiments sulfite reduction has three distinct products: sulfide, reduced thiosulfate S and sulfonate S, each of which is measured for δ^34^*S* and D^33^*S*. Mass and isotopic evolution of residual reactant sulfite is balanced by sulfide and thiosulfate sulfurs at each time point. For the latter, we note that sulfonate sulfur is an approximate redox equivalent of aqueous sulfite (S^5+^; see Vairavamurthy et al., 1993, 1995). There are large fractionations associated with the production of thiosulfate, with the sulfonate and reduced thiosulfate S's separated by nearly 30‰ (both D^33^*S* are slightly positive: Figures 6B, 7A). This is striking given that, over the course of sulfite reduction, the isotopic composition of sulfite becomes only modestly enriched in both δ^34^*S* and Δ^33^*S* (Figure 7).

There are multiple approaches to solve the overall reaction sequence occurring in the sulfite experiment. The first clear possibility is for a stepwise reduction, where trithionate and/or thiosulfate are the necessary intermediates in sulfide production (see above). If trithionate is transiently produced, for every sulfide produced two sulfites would be cycled through trithionate (Equation 4A). For simplicity we assume the two-sulfonate moieties in trithionate would carry identical δ^34^*S* and Δ^33^*S*, given the symmetry of the molecule and identical bonding environment for the oxidized sulfur. If trithionate and thiosulfate formation are similar, the sulfonate sulfurs would be significantly enriched in δ^34^*S* and the reduced S significantly depleted—perhaps near the respective 15 and −15‰ from thiosulfate in sulfite reduction experiments (Figure 6). It is difficult to uniquely predict the composition of the residual sulfite at the time of trithionate production, given the evolving isotopic buffering capacity provided by the size of the extracellular sulfite reservoir. However, the eventual recycling of two of three S atoms would predict a residual reactant sulfite value slightly enriched in δ^34^*S* and depleted in Δ^33^*S* relative to the starting composition, as the only terminal loss (i.e., not recycled back to sulfite) is the production of sulfide. Put differently, the sulfite should close mass balance with the sulfide. This explanation does not fit the data well (see Figure 7B) and fails further when considering the reduced S. If the “step-wise reduction” model is correct, the fate of the reduced S from trithionate is sulfide. For this to be the case, and if we presume that trithionate production would carry a similar fractionation as thiosulfate production, there would have to exist a more than 10‰ *inverse* isotope fractionation associated with S^0^ reduction to sulfide; a process demonstrated to carry little to no fractionation in other systems (Fry et al., 1986). Thus, sulfite reduction in G20 is unlikely to involve a site-specific reduction of trithionate.

Given that trithionate was not detected in our experiments, we now consider the case where thiosulfate is the only reaction intermediate. Thiosulfate production would commence with four electrons transferred onto sulfite, generating an S^0^ equivalent, which can then form a S-S bond with another sulfite molecule, (Equation 4C) (Heunisch, 1977). From there, two possibilities exist. The first would predict that the reduced S is preferentially reduced to sulfide (*S*_2_ *O*^2−^_3_ ⇒ *SO*^2−^_3_ + *HS*^−^), while the sulfonate S is recycled back to the residual sulfite reservoir. However, we observe that the residual sulfite pool remains quite isotopically depleted in δ^34^*S* relative to the sulfonate S (near 0 and +15‰ respectively, Figure 6B). In fact, if this were the mechanism, the stoichiometry of sulfonate S shuttling to sulfite would equal that of sulfide production, such that at minimum, ~50% of the residual sulfite would cycle through thiosulfate by the end of the experiment. Assuming a sulfonate S composition of 15‰, this recycling would pull the residual sulfite composition toward much more enriched values than are observed. Furthermore, the thiosulfate reduction experiment (Figure 4) supports a wholesale and not site-specific reduction pathway. As such, if a step-wise reduction scheme for sulfite is correct, we expect product sulfide to be offset from the bulk thiosulfate composition by approximately 7‰ and along a ^33^λ vector of 0.512 (Figure 5). Early in the experiment sulfide is offset by −7‰ from net thiosulfate (Figure 7), but contracts to −3‰ by the end of the experiment (^33^*S* data is only available for the later two time points, the associated ^33^λ is ~0.5135). These isotopic data suggest that sulfide generation in sulfite reduction experiments is recording wholesale thiosulfate reduction.

For completeness, we also consider that sulfite reduction could follow two *parallel and independent reductions*: *SO*^2−^_3_ ⇒ *HS*^−^ and *SO*^2−^_3_ ⇒ *S*_2_ *O*^2−^_3_. A small fractionation between sulfite and sulfide (^34^ε < 8‰) has been demonstrated in sulfite reduction experiments where only sulfide production is observed (Habicht et al., 1998). Like all solely reductive kinetic processes measured to date, the expectation is for a ^33^λ less than 0.515. This overall scheme appears inconsistent with the data at the later time points, as the δ^34^*S* fractionation between sulfite and sulfide is quite small (<5‰) with an apparent ^33^λ of ~0.519 (Table 3). We specifically term this an apparent ^33^λ given that there may not be a single process relating these pools, and as such, it may not be a process-specific value.

**Figure 8:**
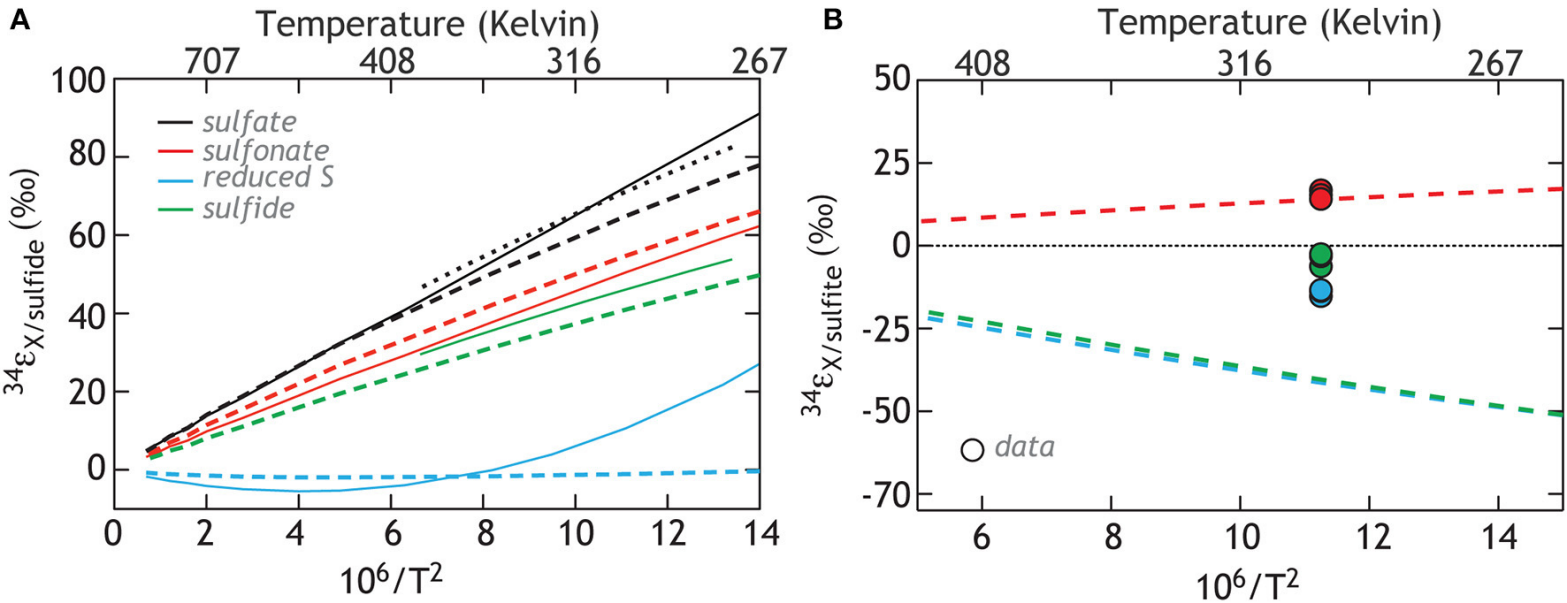
**Calculations of the isotopic equilibrium between sulfate, sulfite, sulfide and the two S sites in thiosulfate**. The color code reflects the species being compared (solid lines: Ohmoto and Lasaga, 1982; Chu et al., 2004, heavy dashed lines: this study, and light (smaller) dashed line: Farquhar et al., 2003). **(A)** The calculations referenced against the composition of sulfide and include published estimates. **(B)** The same calculations, now relative to sulfite, for comparison to data in this study.

Finally, we consider the case where kinetic isotope effects play less of a role, and these observations are controlled by inorganic equilibrium fractionations between different S-bearing moieties. Here we specifically target the two sulfur sites in thiosulfate, sulfite, and sulfide (Figure 8A). The equilibrium calculations yield predictions for the equilibrium fractionation between the different thiosulfate sites and sulfite (Figure 8B). What is immediately clear is the consistency between equilibrium calculations predicting sulfite—sulfonate exchange fractionation and that measured from these experiments. This is consistent with these sites being in equilibrium, reflecting a purely abiological source of sulfonate S, and is supported by early work demonstrating that exchange between these sites is possible (Ames and Willard, 1951). Neither the reduced thiosulfate S or sulfide fit such predictions, indicating that kinetic isotope effects remain central to this system. In fact, the observation that both thiosulfate sulfur sites mass balance back to the starting sulfite is curious if indeed the sulfonate composition is dictated by equilibrium.

Determining a unique solution to how sulfur is transferred and fractionated during sulfite reduction is difficult to ascertain through these experiments alone. Still, given these data, we propose trithionate is no more than a short-lived intermediate, if present at all. It is also difficult to determine if reactions between sulfide and sulfite generate some or all of the thiosulfate, known to occur in abiological mixtures of the two (Heunisch, 1977). We suggest however that, following the initial reduction of sulfite to sulfide and the formation of thiosulfate, the primary metabolic reaction fractionating isotopes is the wholesale reduction of thiosulfate to sulfide. This is similar to that seen in the thiosulfate reduction experiments. The source of thiosulfate requires reduced products and perhaps the scavenging of S^0^ from the DsrAB, which in turn reacts with sulfite, as previously suggested. Shuttling sulfur to a thiosulfate intermediate pool makes some biochemical sense, given the environmental conditions at the beginning of these experiments may not be overly hospitable to a microbial sulfate reducer. The presence of a large ambient sulfite pool imposes significant redox stress on the cell, even though sulfite is a thermodynamically favorable electron acceptor. If the organism is capable of generating a more stable reservoir of electron acceptor, such as thiosulfate, this may be optimal. Indeed, this is what we propose here: sulfite is shuttled to thiosulfate and then reduced to sulfide. This could be happening in parallel to some direct sulfide production from sulfite. Revisiting the role of sulfite during sulfate reduction, it will be critical in future works to determine the intra-cellular concentrations of sulfite, and whether they can approach those of our media (10 mM).

## Interpretations in light of MSR biochemistry

Our overall motivation and emphasis rests with characterizing the isotopic consequences of sulfite reduction within microbial sulfate reduction network. This is grounded in the central role that sulfite plays within MSR, and energy conservation in particular (Bradley et al., 2011). Understanding what controls the shuttling of sulfite to particular intermediates (possibilities including trithionate, thiosulfate, zero-valent sulfur, or sulfide) and the isotopic consequences of the possible shunts (both the intrinsic fractionation of each reaction and mass balance consequences at the cellular scale) are tractable through the types of experiments presented here. We note that we are not the first to engage in such a pursuit, and our experimental protocol is adopted from earlier works. Harrison and Thode (1958), for instance, found that *Desulfovibrio desulfuricans* reduced sulfite at the same rate and associated isotope fractionation as during sulfate reduction. Other studies since that time have targeted sulfite reduction, some of which suggest the direct production of sulfide while others illustrate the importance of intermediates (Harrison and Thode, 1958; Kaplan et al., 1963; Krouse et al., 1968; Fitz and Cypionka, 1990; Sass et al., 1992; Habicht et al., 1998; Smock et al., 1998). In an ideal case, the reduction of sulfoxy anion intermediates in experiments where they are the lone electron acceptor would perfectly mimic the *in vivo* biochemical reactions present when grown on sulfate. Furthermore, we extend the insights gleaned from our experiments from major isotope information by including ^33^*S* and, where necessary, site-specific isotope measurements.

The *in vivo* fate of sulfite during MSR was presumed to most often proceed via a six-electron reduction to sulfide, followed by hydrogen sulfide diffusion out of the cell (Peck et al., 1982). Recent work demonstrates that sulfite is reduced stepwise (Drake and Akagi, 1977a, 1978; Oliveira et al., 2008; Venceslau et al., 2010), likely in two-electron increments (detailed below). As an aside, some studies show that sulfide can be reoxidized to sulfate during active MSR, arguably via reversal of the MSR pathway (Trudinge and Chambers, 1973; Eckert et al., 2011; Holler et al., 2011). However, the explicit biotic or abiotic mediation of such sulfide re-oxidation is unclear. One alternative to sulfite reduction is re-oxidation to sulfate—a flux detected by oxygen isotope incorporation studies (Aharon and Fu, 2000, 2003; Farquhar et al., 2008; Mangalo et al., 2008; Wankel et al., 2013). Another potential fate for sulfite, and one that is not well studied, is to undergo partial reduction and reside within any one of a number of thionates, such as trithionate or thiosulfate (Kobayashi et al., 1969; Drake and Akagi, 1977b, 1978). Finally, sulfite may act as both electron donor and acceptor in the inorganic fermentation reaction known as “disproportionation” (Finster, 2008).

The evidence for thionates during MSR dates back more than 45 years. Thionate production was observed in in vitro studies (Findley and Akagi, 1969; Kobayashi et al., 1969, 1972; Lee and Peck, 1971; Lee et al., 1973), as well as in *in vivo* experiments under non-traditional growth conditions of electron donor and/or sulfite availability (Fitz and Cypionka, 1990; Sass et al., 1992; Akgaki, 1995; Broco et al., 2005; Davidson et al., 2009). Despite these findings, the overall significance of the trithionate pathway to net MSR was unclear. Some argue against its presence on the basis of evidence from *in vivo* ^35^S labeling studies (Chambers and Trudinger, 1975), whereas others argue in opposition to the trithionate pathway because the reduction of trithionate and thiosulfate did not appear to be coupled to proton translocation (Peck et al., 1982). It was then suggested that the main benefit of thionate production/reduction is the regeneration of sulfite (Bradley et al., 2011), which can impose an undue redox stress on the cell if present at high concentration (i.e., excess of a reactive intermediate that can be readily oxidized or reduced, depending on the intracellular environmental E_h_), despite the fact that it is a key intermediate in MSR.

Two simple conclusions arise from the observations to-date. First, thionate production and consumption is possible given the known MSR biochemistry (Akagi et al., 1994; Parey et al., 2010). Second, the energetic conditions driving these reactions (Thauer et al., 1977), biochemical consequences and resultant isotopic fractionations remain unclear (Bradley et al., 2011). It is within the details of existing biochemical research that we come to understand the production of the sulfur intermediates. The major redox-active proteins in this system are APS reductase (APSr), dissimilatory sulfite reductase (DsrAB), and sulfur transfer protein (DsrC). APSr is not thought to be involved in the production of thionates, whereas the structural and catalytic properties of DsrAB suggest that it can aid in the production of thionates (Parey et al., 2010). In both δ-Proteobacterial and Archeal forms, DsrAB consists of the two major subunits DsrA and DsrB, arranged as a α_2_β_2_ homodimer (Oliveira et al., 2008, 2011; Parey et al., 2010). Each αβ subunit contains a sirohydrochlorin, which upon metalation with Fe, becomes a siroheme active site where sulfite can bind and receive electrons. Two iron—sulfur clusters are proximal and assist in the transfer of electrons from an as yet unidentified cytoplasmic electron donor, lending DsrAB the capacity to reduce sulfite first to a S^2+^ equivalent and possibly then to a S^0^ equivalent, via two two-electron transfer steps (Oliveira et al., 2008). The most crucial insight derived from the DsrAB/C crystal structures was the recognition that DsrC is an independent weakly bound second protein (Oliveira et al., 2008; Parey et al., 2010). The physical association of DsrC with DsrAB is very close (Pierik et al., 1992), such that the redox active C-terminus is capable of binding the partially reduced sulfur (2+ or 0 valent) from the siroheme, and transporting it to the DsrMKJOP for terminal reduction to sulfide (Mander et al., 2005; Oliveira et al., 2008; Venceslau et al., 2010, 2014)—this ultimately couples net sulfite reduction to energy conservation (Venceslau et al., 2010, 2014). As discussed elsewhere (Bradley et al., 2011), the relative efficiencies of DsrAB and DsrC may dictate the availability of SO^2−^_3_, S^2+^, and S^0^ to thionate production. That is, when DsrAB produces S^2+^ and S^0^ equivalents faster than DsrC can extract the DsrAB-bound sulfurs, then trithionate and thiosulfate may be produced by residual sulfite scavenging of the partially reduced (S^2+^ and S^0^) intermediates from the DsrAB active-site. The rate of S^2+^ and S^0^ production and reduction is then related to electron delivery to DsrAB relative to DsrC (assuming non-limiting sulfite), rather than the saturation of the DsrAB active site (Bradley et al., 2011).

An additional explanation for the production and consumption of thionates calls upon a different suite of enzymes to catalyze these reactions. If true, this hypothesis requires future biochemical identification of one (and up to three) novel enzyme (reviewed in Bradley et al., 2011). Despite this shortcoming, isotopes provide a means of distinguishing partial pathways that are shared between metabolic regimes from those that are unique. Isotope fractionations may make differentiation of various metabolic pathways possible in the absence of strict biochemical information. Finally, thermodynamic calculations predict that the conversion of sulfite to trithionate or thiosulfate and the reduction of either sulfite or the thionates to sulfide are exergonic reactions, whilst the reduction of sulfate to sulfite is endergonic (Thauer et al., 1977). This is consistent with biochemical data, which suggests that energy conservation in MSR is downstream of sulfite, and associated with the reduction of S^2+^ and S^0^ valent sulfur to sulfide, via the oxidation of reduced DsrC.

It is important to appreciate that performing sulfite reduction experiments with whole cells and live cultures is not a perfectly analogous system to the operation of DsrAB/C *in vivo* within a fully constituted sulfate reducer. Thus, we discuss below the range of possibilities to help us hone in on the particular reactions and fractionation factors that are expressed, and are aware of the limits of this approach. Our characterization of sulfate reduction by our model organism (G20), using two different electron donors is consistent with previous works. G20 produces small ^34^ε fractionations while operating at high csSRR. These data also extend our picture of ^33^*S* fractionation from sulfate reduction at small δ^34^*S* and are consistent with the composite ^33^λ vs. δ^34^*S* relation from previous works (citations in Figure 3).

Thiosulfate reduction presented a number of possible reduction schemes, however our results suggest a simple, wholesale reduction to sulfide. There are interesting implications at the enzyme level if this is true. Specifically, once the thiosulfate is bound to the active site, both sulfurs are reduced to the valence where DsrC can bind and reduce each to sulfide. That is, they must remain bound to or closely associated with the active site of the host enzyme such that loss of either S back to the bulk intracellular solution is minimized. Differentiating reaction pathways at such spatial and temporal scales will require knowledge of the enzymes involved, the electron delivery scheme, and enzyme-substrate geometry throughout the reduction process.

The *in vivo* reduction of sulfite proved to be quite complex in G20. Although the measured products are thiosulfate and sulfide (in a fixed ratio, see Table 3), it is possible that trithionate is the first intermediate, but below analytical detection due to a very short residence time. The most parsimonious interpretation of the sulfite-thiosulfate-sulfide system is the transformation of sulfite to thiosulfate, which is then quantitatively reduced to sulfide. This does not have to be the only means by which sulfide is generated, but may carry the largest isotopic and mass consequence. The largest fractionation here is associated with the reductive formation of thiosulfate from sulfite, however this isotope effect is largely lost upon the ensuing reduction to sulfide. We reach this interpretation based on the parallel thiosulfate reduction experiments with the same organism. Further, the role of DsrAB/C in this system is also unclear. The enzyme system may be acting independently or in parallel with separate thiosulfate forming and reducing enzymes or performing all reactions independently (see Discussion in Bradley et al., 2011). The difference in fractionations we observed between sulfate vs. thiosulfate reduction experiments may simply relate to differences in net metabolic rate with the same enzymatic machinery (c.f. Leavitt et al., 2013), or may indicate the variable behavior of a given protein, or operation of different enzymes. Differentiating between these scenarios is beyond the scope of this study, but critical to ultimately understanding these reactions.

## Conclusions

This collection of observations has clear implications for how we interpret MSR isotope fractionation experiments and S isotope fractionations in natural systems. The cycling of sulfur intermediates like sulfite and thiosulfate involves more than single reaction step and can carry significant isotope effects—fractionation that may be hidden in light of a cellular scale reaction like MSR. This is especially true during thiosulfate production in the sulfite reduction experiments. Recent work predicts both sulfite and thiosulfate to have played prominent roles as an electron acceptors early in Earth history, when sulfate concentrations in the oceans were likely two-orders of magnitude less than the present 28 mM (Halevy, 2013). A careful revisiting of the enzymes involved in sulfite and thiosulfate reduction reactions is a target for future research. As the key enzymes come to be better understood, the similarities and differences with companion processes like sulfur disproportionation will also become more clear, both in their biochemical and isotopic fractionation capacities. As presented above, the production of thiosulfate from sulfite can induce an apparent 30‰ intermolecular fractionation, even before thiosulfate reduction (or disproportionation) begins. How we view net isotope fractionation between preserved sulfate and sulfide minerals in modern or ancient environments, where a broad array of reactions are possible prior to S anion preservation, must consider a suite of S intermediates, adding further nuance to isotope geochemical box models. Future microbiological work will help to define how and when certain reactions are catalyzed and by which enzymes. Once MSR reactions are understood at that level, and with complementary data from oxidation and disproportionation reactions, a more biochemically correct framework can be constructed in which the most parsimonious environmental interpretations may be possible.

### Conflict of interest statement

The authors declare that the research was conducted in the absence of any commercial or financial relationships that could be construed as a potential conflict of interest.

## Footnotes

^1^It is critical to demonstrate the closure of isotopic mass balance. Relative proportions are noted as:

$$f_{sulfide}={\left[sulfide\right]}_{t\;=\;n}/{\left[sulfite\right]}_{t\;=\;0}$$

and

$$f_{thiosulfate}={\left[thiosulfate\right]}_{t\;=\;n}/{\left[sulfite\right]}_{t\;=\;0}.$$

We note that two-component mixing is nonlinear in δ34*S* − Δ^33^*S* (see Figure 7B). We therefore convert compositions back to ^3*x*^*R* and solve for both ^33^*R* and ^34^*R* independently (where *R* is the ratio of the target pool normalized to the initial sulfite), such that for any given time point $\begin{array}{l} (t=n){:}^{3x}R_{sulfite,\;t\;=\;0}=\;([1-f_{sulfide},-f_{thiosulfate},]\times^{3x}R_{sulfite,\;t\;=\;n})+\\ \left(f_{sulfide},\times^{3x}R_{sulfide,\;t\;=\;n})+(0.5f_{thiosulfate},\times^{3x}R_{sulfonate-S,\;t\;=\;n}\right)\\ +(0.5f_{thiosulfate},\times^{3x}R_{sulfane-S,\;t\;=\;n}) \end{array}$. The difference between the bulk composition of the experiment at time zero (constituting total S in the experiment) and our summed pools of directly quantified S at any given time point is within 1σ of one another, once analytical errors (for *f* and δ) are propagated. This harkens back to the essential nature of measuring the concentration of intermediate species and closure of overall mass balance. Under these tight constraints, we are able to interpret the data presented in Figure 7.

## References

[B1] Aharon P. Fu B. S. (2000). Microbial sulfate reduction rates and sulfur and oxygen isotope fractionations at oil and gas seeps in deepwater Gulf of Mexico. Geochim. Cosmochim. Acta 64, 233–246 10.1016/S0016-7037(99)00292-6

[B2] Aharon P. Fu B. S. (2003). Sulfur and oxygen isotopes of coeval sulfate-sulfide in pore fluids of cold seep sediments with sharp redox gradients. Chem. Geol. 195, 201–218 10.1016/S0009-2541(02)00395-9

[B3] Akagi J. Drake H. Kim J. Gevertz D. (1994). Thiosulfate and trithionate reductases. Methods Enzymol. 43, 260–270 10.1016/0076-6879(94)43019-5

[B4] Akgaki J. (1995). Respiratory sulfate reduction, in Sulfate-Reducing Bacteria, ed Barton L. (New York, NY: Plenum Press), 89–111.

[B5] Alroy J. Aberhan M. Bottjer D. J. Foote M. Fursich F. T. Harries P. J.. (2008). Phanerozoic trends in the global diversity of marine invertebrates. Science 321, 97–100. 10.1126/science.1156963 18599780

[B6] Ames D. P. Willard J. E. (1951). The Kinetics of the exchange of sulfur between thiosuflate and sulfite. J. Am. Chem. Soc. 73, 164–172 10.1021/ja01145a059

[B7] Berner R. A. Canfield D. E. (1989). A new model for atmospheric oxygen over Phanerozoic time. Am. J. Sci. 289, 333–361. 10.2475/ajs.289.4.333 11539776

[B8] Bigeleisen J. Mayer M. G. (1947). Calculation of equilibrium constants for isotopic exchange reactions. J. Chem. Phys. 15, 261–267. 10.1063/1.174649219161313

[B9] Bowles M. W. Mogollon J. M. Kasten S. Zabel M. Hinrichs K. U. (2014). Global rates of marine sulfate reduction and implications for sub-sea-floor metabolic activities. Science 344, 889–891. 10.1126/science.1249213 24812207

[B10] Bradley A. S. Leavitt W. D. Johnston D. T. (2011). Revisiting the dissimilatory sulfate reduction pathway. Geobiology 9, 446–457. 10.1111/j.1472-4669.2011.00292.x 21884365

[B11] Broco M. Rousset M. Oliveira S. Rodrigues-Pousada C. (2005). Deletion of flavoredoxin gene in *Desulfovibrio gigas* reveals its participation in thiosulfate reduction. FEBS Lett. 579, 4803–4807. 10.1016/j.febslet.2005.07.044 16099456

[B12] Brunner B. Bernasconi S. M. (2005). A revised isotope fractionation model for dissimilatory sulfate reduction in sulfate reducing bacteria. Geochim. Cosmochim. Acta 69, 4759–4771 10.1016/j.gca.2005.04.015

[B13] Canfield D. E. (2001a). Biogeochemistry of sulfur isotopes. Stable Isotope Geochem. 43, 607–636 10.2138/gsrmg.43.1.607

[B14] Canfield D. E. (2001b). Isotope fractionation by natural populations of sulfate-reducing bacteria. Geochim. Cosmochim. Acta 65, 1117–1124. 10.1016/S0016-7037(00)00584-611541664

[B15] Canfield D. E. (2004). The evolution of the Earth surface sulfur reservoir. Am. J. Sci. 304, 839–861 10.2475/ajs.304.10.839

[B16] Canfield D. E. Desmarais D. J. (1994). Biogeochemical cycles of carbon, sulfur, and free oxygen in a microbial mat. Geochim. Cosmochim. Acta 57, 1044–1044. 10.1016/0016-7037(94)90526-6 11537735

[B17] Canfield D. E. Farquhar J. Zerkle A. L. (2010). High isotope fractionations during sulfate reduction in a low-sulfate euxinic ocean analog. Geology 38, 415–418 10.1130/G30723.1

[B18] Canfield D. E. Raiswell R. (1999). The evolution of the sulfur cycle. Am. J. Sci. 299, 697–723 10.2475/ajs.299.7-9.697

[B19] Chambers L. A. Trudinger P. A. (1975). Are thiosulfate and trithionate intermediates in dissimilatory sulfate reduction? J. Bacteriol. 123, 36–40. 1141200 10.1128/jb.123.1.36-40.1975 PMC235688

[B20] Chambers L. A. Trudinger P. A. Smith J. W. Burns M. S. (1975). Fractionation of sulfur isotopes by continuous cultures of *Desulfovibrio desulfuricans*. Can. J. Microbiol. 21, 1602–1607. 10.1139/m75-234 1201506

[B21] Chu X. Ohmoto H. Cole D. R. (2004). Kinetics of sulfur isotope exchange between aqueous sulfide and thiosulfate involving intra- and intermolecular reactions at hydrothermal conditions. Chem. Geol. 211, 217–235 10.1016/j.chemgeo.2004.06.013

[B22] Cline J. D. (1969). Spectrophotometric determination of hydrogen sulfide in natural waters. Limnol. Oceanogr. 14, 454–465 10.4319/lo.1969.14.3.0454

[B23] Davidson M. M. Bisher M. E. Pratt L. M. Fong J. Southam G. Pfiffner S. M.. (2009). Sulfur isotope enrichment during maintenance metabolism in the thermophilic sulfate-reducing bacterium *Desulfotomaculum putei*. Appl. Environ. Microbiol. 75, 5621–5630. 10.1128/AEM.02948-08 19561180 PMC2737900

[B24] Detmers J. Bruchert V. Habicht K. S. Kuever J. (2001). Diversity of sulfur isotope fractionations by sulfate-reducing prokaryotes. Appl. Environ. Microbiol. 67, 888–894. 10.1128/AEM.67.2.888-894.2001 11157259 PMC92663

[B25] Drake H. L. Akagi J. M. (1977a). Bisulfite reductase of *Desulfovibrio-vulgaris* – explanation for product formation. J. Bacteriol. 132, 139–143. 914772 10.1128/jb.132.1.139-143.1977 PMC221837

[B26] Drake H. L. Akagi J. M. (1977b). Characterization of a novel thiosulfate forming enzyme isolated from *Desulfovibrio-vulgaris*. J. Bacteriol. 132, 132–138. 199572 10.1128/jb.132.1.132-138.1977 PMC221836

[B27] Drake H. L. Akagi J. M. (1978). Dissimilatory reduction of bisulfite by *Desulfovibrio-vulgaris*. J. Bacteriol. 136, 916–923. 721780 10.1128/jb.136.3.916-923.1978 PMC218525

[B28] Eckert T. Brunner B. Edwards E. A. Wortmann U. G. (2011). Microbially mediated re-oxidation of sulfide during dissimilatory sulfate reduction by *Desulfobacter latus*. Geochim. Cosmochim. Acta 75, 3469–3485 10.1016/j.gca.2011.03.034

[B29] Farquhar J. Canfield D. E. Masterson A. Bao H. Johnston D. (2008). Sulfur and oxygen isotope study of sulfate reduction in experiments with natural populations from Faellestrand, Denmark. Geochim. Cosmochim. Acta 72, 2805–2821 10.1016/j.gca.2008.03.013

[B30] Farquhar J. Johnston D. T. Wing B. A. Habicht K. S. Canfield D. E. Airieau S. (2003). Multiple sulphur isotopic interpretations of biosynthetic pathways: implications for biological signatures in the sulphur isotope record. Geobiology 1, 27–36 10.1046/j.1472-4669.2003.00007.x

[B31] Farquhar J. Wing B. A. (2003). Multiple sulfur isotopes and the evolution of the atmosphere. Earth Planet. Sci. Lett. 213, 1–13. 10.1016/S0012-821X(03)00296-623836655

[B32] Findley J. Akagi J. (1969). Evidence for thiosulfate formation during sulfite reduction by *Desulfovibrio vulgaris*. Biochem. Biophys. Res. 36, 266–271. 10.1016/0006-291X(69)90324-6 5799644

[B33] Finster K. (2008). Microbiological disproportionation of inorganic sulfur compounds. J. Sulfur Chem. 29, 281–292 10.1080/17415990802105770

[B34] Fitz R. M. Cypionka H. (1989). A study on electron transport driven proton translocation in *Desulfovibrio - desulfuricans*. Arch. Microbiol. 152, 369–376 10.1007/BF00425175 2476099

[B35] Fitz R. M. Cypionka H. (1990). Formation of thiosulfate and trithionate during sulfite reduction by washed cells of *Desulfovibrio desulfuricans*. Arch. Microbiol. 154, 400–406 10.1007/BF00276538

[B36] Forrest J. Newman L. (1977). Ag 110 microgram sulfate analysis for short time resolution of ambient levels of sulfur aerosols. Anal. Chem. 49, 1579–1584. 10.1021/ac50019a030 900494

[B37] Fry B. Cox J. Gest H. Hayes J. M. (1986). Discrimination between 34S and 32S during bacterial metabolism of inorganic sulfur compounds. J. Bacteriol. 165, 328–330. 3941049 10.1128/jb.165.1.328-330.1986 PMC214413

[B38] Goldhaber M. B. Kaplan I. R. (1975). Controls and consequecnes of sulfate reduction rates in recent marine sediments. Soil Sci. 119, 42–55 10.1097/00010694-197501000-00008

[B39] Habicht K. S. Canfield D. E. (2001). Isotope fractionation by sulfate-reducing natural populations and the isotopic composition of sulfide in marine sediments. Geology 29, 555–558 10.1130/0091-7613(2001)029%3C0555:IFBSRN%3E2.0.CO;2

[B40] Habicht K. S. Canfield D. E. Rethmeier J. (1998). Sulfur isotope fractionation during bacterial reduction and disproportionation of thiosulfate and sulfite. Geochim. Cosmochim. Acta 62, 2585–2595. 10.1016/S0016-7037(98)00167-7 11541664

[B41] Habicht K. S. Gade M. Thamdrup B. Berg P. Canfield D. E. (2002). Calibration of sulfate levels in the Archean Ocean. Science 298, 2372–2374. 10.1126/science.1078265 12493910

[B42] Habicht K. S. Salling L. L. Thamdrup B. Canfield D. E. (2005). Effect of low sulfate concentrations on lactate oxidation and isotope Fractionation during sulfate reduction by *Archaeoglobus fulgidus* strain Z. Appl. Environ. Microbiol. 71, 3770–3777. 10.1128/AEM.71.7.3770-3777.2005 16000788 PMC1168999

[B43] Halevy I. (2013). Production, preservation, and biological processing of mass-independent sulfur isotope fractionation in the Archean surface environment. Proc. Natl. Acad. Sci. U.S.A. 110, 17644–17649. 10.1073/pnas.1213148110 23572589 PMC3816473

[B44] Halevy I. Peters S. E. Fischer W. W. (2012). Sulfate burial constraints on the Phanerozoic sulfur cycle. Science 337, 331–334. 10.1126/science.1220224 22822147

[B45] Harrison A. G. Thode H. G. (1958). Mechanism of the bacterial reduction of sulphate from isotope fractionation studies. Trans. Faraday Soc. 54, 84–92 10.1039/tf9585400084

[B46] Haschke R. H. Campbell L. L. (1971). Thiosulfate reductase of *Desulfovibrio - vulgaris*. J. Bacteriol. 106, 603–609. 5573735 10.1128/jb.106.2.603-607.1971 PMC285136

[B47] Hatchikian E. C. (1975). Purification and proteries of thiosulfate reductase from *Desulfovibrio - gigas*. Arch. Microbiol. 105, 249–256. 10.1007/BF00447143 242299

[B48] Hayes J. M. (2001). Fractionation of carbon and hydrogen isotopes in biosynthetic processes. Stable Isotope Geochem. 43, 225–277 10.2138/gsrmg.43.1.225

[B49] Hayes J. M. Waldbauer J. R. (2006). The carbon cycle and associated redox processes through time. Philos. Trans. R. Soc. B Biol. Sci. 361, 931–950. 10.1098/rstb.2006.1840 16754608 PMC1578725

[B50] Heunisch G. W. (1977). Stoichiometry of reaction of sulfites with hydrogen sulfide ion. Inorg. Chem. 16, 1411–1413. 10.1021/ic50172a0336284747

[B51] Holland H. D. (1973). Systematics of isotopic composition of sulfur in oceans during Phanerozoic and its implications for atmospheric oxygen. Geochim. Cosmochim. Acta 37, 2605–2616 10.1016/0016-7037(73)90268-8

[B52] Holler T. Wegener G. Niemann H. Deusner C. Ferdelman T. G. Boetius A.. (2011). Carbon and sulfur back flux during anaerobic microbial oxidation of methane and coupled sulfate reduction. Proc. Natl. Acad. Sci. U.S.A. 108, E1484–E1490. 10.1073/pnas.1106032108 22160711 PMC3248532

[B53] Johnston D. T. (2011). Multiple sulfur isotopes and the evolution of Earth's surface sulfur cycle. Earth-Sci. Rev. 106, 161–183 10.1016/j.earscirev.2011.02.003

[B54] Johnston D. T. Farquhar J. Canfield D. E. (2007). Sulfur isotope insights into microbial sulfate reduction: When microbes meet models. Geochim. Cosmochim. Acta 71, 3929–3947 10.1016/j.gca.2007.05.008

[B55] Johnston D. T. Farquhar J. Wing B. A. Kaufman A. Canfield D. E. Habicht K. S. (2005). Multiple sulfur isotope fractionations in biological systems: a case study with sulfate reducers and sulfur disproportionators. Am. J. Sci. 305, 645–660 10.2475/ajs.305.6-8.645

[B56] Jorgensen B. B. (1982). Mineralization of organic matter in the sea bed - the role of sulfate reduction. Nature 296, 643–645 10.1038/296643a0

[B57] Kaplan I. R. Emery K. O. Rittenberg S. C. (1963). The distribution and isotopic abundance of sulphur in recent marine sediments off southern California. Geochim. Cosmochim. Acta 27:297. 10.1016/0016-7037(63)90074-722352384

[B58] Kaplan I. R. Rittenberg S. C. (1964). Microbiological fractionation of sulphur isotopes. J. Gen. Microbiol. 34:195. 10.1099/00221287-34-2-195 14135528

[B110] Kelly D. P. Wood A. P. (1994). Synthesis and determination of thiosulfate and polythionates. Meth. Enzymol. 243, 475–501.

[B59] Kobayashi K. Tachibana S. Ishimoto M. (1969). Intermediary formation of trithionate in sulfite reduction by a sulfate-reducing bacterium. J. Biochem. (Tokyo) 65:155. 5771706

[B60] Kobayashi K. Takahashi E. Ishimoto M. (1972). Biochemical studies on sulfate-reducing bacteria. XI. Purification and some properties of sulfite reductase, desulfoviridin. J. Biochem. (Tokyo) 72, 879–887. 4644321 10.1093/oxfordjournals.jbchem.a129982

[B61] Krouse H. R. McCready G. L. Husain S. A. Campbell J. N. (1968). Sulfur isotope fractionation and kinetic studies of sulfite reduction in growing cells of *Salmonella heidelberg*. Biophys. J. 8, 109–120. 10.1016/S0006-3495(68)86478-1 5641397 PMC1367362

[B62] Kurtz A. C. Kump L. R. Arthur M. A. Zachos J. C. Paytan A. (2003). Early Cenozoic decoupling of the global carbon and sulfur cycles. Paleoceanography 18, 1090–1104 10.1029/2003PA000908

[B63] Leavitt W. (2014). On the Mechanisms of Sulfur Isotope Fractionation During Microbial Sulfate Reduction. Earth and Planetary Sciences. Cambridge, MA: Harvard University, ProQuest.

[B64] Leavitt W. D. Bradley A. S. Halevy I. Johnston D. T. (2013). Influence of sulfate reduction rates on the Phanerozoic sulfur isotope record. Proc. Natl. Acad. Sci. U.S.A. 110, 11244–11249. 10.1073/pnas.1218874110 23733944 PMC3710818

[B65] Lee J. Peck H. (1971). Purification of enzyme reducing bisulfite to trithionate from *Desulfovibrio gigas* and its identification as desulfoviridin. Biochem. Biophys. Res. Commun. 45, 583–589. 10.1016/0006-291X(71)90457-8 5128167

[B66] Lee J. Yi C. LeGall J. Peck H. (1973). Isolation of a new pigment, desulforubidin, from *Desulfovibrio desulfuricans* (Norway strain) and its role in sulfite reduction. J. Bacteriol. 115, 453–455. 4717523 10.1128/jb.115.1.453-455.1973 PMC246260

[B67] Mander G. Weiss M. Hedderich R. Kahnt J. Ermler U. Warkentin E. (2005). X-ray structure of the [gamma]-subunit of a dissimilatory sulfite reductase: fixed and flexible C-terminal arms. FEBS Lett. 579, 4600–4604. 10.1016/j.febslet.2005.07.029 16098517

[B68] Mangalo M. Einsiedl F. Meckenstock R. U. Stichler W. (2008). Influence of the enzyme dissimilatory sulfite reductase on stable isotope fractionation during sulfate reduction. Geochim. Cosmochim. Acta 72, 1513–1520 10.1016/j.gca.2008.01.006

[B69] Miertus S. Scrocco E. Tomasi J. (1981). Electrostatic interaction of a solute with a continuum – a direct utilization of ab initio molecular potentials for the prevision of solvent effects. Chem. Phys. 55, 117–129 10.1016/0301-0104(81)85090-2

[B70] Miertus S. Tomasi J. (1982). Approximate evaluations of the electrostatic free energy and internal energy changes in solution processes. Chem. Phys. 65, 239–245 10.1016/0301-0104(82)85072-6

[B71] Miller M. F. (2002). Isotopic fractionation and the quantification of O-17 anomalies in the oxygen three-isotope system: an appraisal and geochemical significance. Geochim. Cosmochim. Acta 66, 1881–1889 10.1016/S0016-7037(02)00832-3

[B72] Moore L. R. Rocap G. Chisholm S. W. (1998). Physiology and molecular phylogeny of coexisting Prochlorococcus ecotypes. Nature 393, 464–467. 10.1038/30965 9624000

[B73] Nakai N. Jensen M. L. (1964). The kinetic isotope effect in the bacterial reduction and oxidation of sulfur. Geochim. Cosmochim. Acta 28, 1893–1912 10.1016/0016-7037(64)90136-X

[B74] Nakatsuk W. Akagi J. M. (1969). Thiosulfate reductase isolated from Desulfotomaculum nigrificans. J. Bacteriol. 98, 429–435. 5784203 10.1128/jb.98.2.429-433.1969 PMC284833

[B75] Newton G. L. Fahey R. C. (1995). Determination of biothiols by bromobimane labeling and high performance liquid chromotography. Biothiols Pt A 251, 148–166. 7651194 10.1016/0076-6879(95)51118-0

[B76] Ohmoto H. Lasaga A. C. (1982). Kinetics of reactions between aqueous sulfates and sulfides in hydrothermal systems. Geochim. Cosmochim. Acta 46, 1727–1745 10.1016/0016-7037(82)90113-2

[B77] Oliveira T. F. Franklin E. Afonso J. P. Khan A. R. Oldham N. J. Pereira I. A. C.. (2011). Structural insights into dissimilatory sulfite reductases: structure of desulforubidin from *Desulfomicrobium norvegicum*. Front. Microbiol. 2:71. 10.3389/fmicb.2011.00071 21833321 PMC3153041

[B78] Oliveira T. F. Vonrhein C. Matias P. M. Venceslau S. S. Pereira I. A. C. Archer M. (2008). The crystal structure of *Desulfovibrio vulgaris* dissimilatory sulfite reductase bound to DsrC provides novel insights into the mechanism of sulfate respiration. J. Biol. Chem. 283, 34141–34149. 10.1074/jbc.M805643200 18829451 PMC2662231

[B79] Ono S. H. Keller N. S. Rouxel O. Alt J. C. (2012). Sulfur-33 constraints on the origin of secondary pyrite in altered oceanic basement. Geochim. Cosmochim. Acta 87, 323–340 10.1016/j.gca.2012.04.016

[B80] Parey K. Warkentin E. Kroneck P. M. H. Ermler U. (2010). Reaction cycle of the dissimilatory sulfite reductase from *Archaeoglobus fulgidus*. Biochemistry 49, 8912–8921. 10.1021/bi100781f 20822098

[B81] Peck H. D. (1959). The ATP-dependent reduction of sulfate with hydrogen in extracts of *Desulfovibrio desulfuricans*. Proc. Natl. Acad. Sci. U.S.A. 45, 701–708. 10.1073/pnas.45.5.701 16590430 PMC222620

[B82] Peck H. D. (1962). Role of Adenosine 5′ phosphosulfate in reduction of sulfate to sulfite by *Desulfovibrio desulfuricans*. J. Biol. Chem. 237, 198–203. 14484820

[B83] Peck H. D. Legall J. Vanbeeumen J. (1982). Biochemistry of dissimilatory sulfate reduction. Philos. Trans. R. Soc. Lond. B Biol. Sci. 298, 443–466. 10.1098/rstb.1982.0091 6127735

[B84] Pierik A. J. Duyvis M. G. van Helvoort J. M. Wolbert R. B. Haven W. R. (1992). The third subunit of desulfoviridin-type dissimilatory sulfite reductases. Eur. J. Biochem. 205, 111–115. 10.1111/j.1432-1033.1992.tb16757.x 1555572

[B85] Price M. Ray J. Wetmore K. Kuehl J. Bauer S. Deutschbauer A.. (2014). The genetic basis of evergy conservation in the sulfate-reducing bacterium *Desulfovibrio alaskensis* G20. Front. Microbiol. 5:577. 10.3389/fmicb.2014.00577 25400629 PMC4215793

[B86] Rabus R. Hansen T. A. Widdel F. (2006). Dissimilatory sulfate- and sulfur-reducing prokaryotes. Prokaryotes 2, 659–768 10.1007/0-387-30742-7_22

[B87] Rees C. E. (1973). Steady-state model for sulfur isotope fractionationin bacterial reduction processes. Geochim. Cosmochim. Acta 37, 1141–1162 10.1016/0016-7037(73)90052-5

[B88] Sass H. Steuber J. Kroder M. Kroneck P. Cypionka H. (1992). Formation of thionates by fresh-water and marine strains of sulfate-reducing bacteria. Arch. Microbiol. 158, 418–421 10.1007/BF00276302

[B89] Scott A. P. Radom L. (1996). Harmonic vibrational frequencies: an evaluation of Hartree-Fock, Moller-Plesset, quadratic configuration interaction, density functional theory, and semiempirical scale factors. J. Phys. Chem. 100, 16502–16513 10.1021/jp960976r

[B90] Shirodkar S. Reed S. Romine M. Saffarini D. (2011). The octahaem SirA catalyses dissimilatory sulfite reduction in *Shewanella oneidensis* MR-1. Environ. Microbiol. 13, 108–115. 10.1111/j.1462-2920.2010.02313.x 21199252

[B91] Sim M. S. Bosak T. Ono S. (2011a). Large sulfur isotope fractionation does not require disproportionation. Science 333, 74–76. 10.1126/science.1205103 21719675

[B92] Sim M. S. Ono S. Bosak T. (2012). Effects of iron and nitrogen limitation on sulfur isotope fractionation during microbial sulfate reduction. Appl. Environ. Microbiol. 78, 8368–8376. 10.1128/AEM.01842-12 23001667 PMC3497358

[B93] Sim M. S. Ono S. Donovan D. Templer S. P. Bosak T. (2011b). Effect of electron donors on the fractionation of sulfur isotopes by a marine *Desulfovibrio* sp. Geochim. Cosmochim. Acta 75, 4244–4259 10.1016/j.gca.2011.05.021

[B94] Smock A. M. Bottcher M. E. Cypionka H. (1998). Fractionation of sulfur isotopes during thiosulfate reduction by *Desulfovibrio desulfuricans*. Arch. Microbiol. 169, 460–463. 10.1007/s002030050597 9560428

[B95] Strauss H. (1997). The isotopic composition of sedimentary sulfur through time. Palaeogeogr. Palaeoclimatol. Palaeoecol. 132, 97–118 10.1016/S0031-0182(97)00067-9

[B96] Strauss H. (1999). Geological evolution from isotope proxy signals - sulfur. Chem. Geol. 161, 89–101 10.1016/S0009-2541(99)00082-0

[B97] Thauer R. K. Jungermann K. Decker K. (1977). Energy conservation in chemotrophic anaerobic bacteria. Bacteriol. Rev. 41, 100–180. 860983 10.1128/br.41.1.100-180.1977 PMC413997

[B98] Thode H. G. Monster J. Dunford H. B. (1961). Sulphur isotope geochemistry. Geochim. Cosmochim. Acta 25, 159–174 10.1016/0016-7037(61)90074-6

[B99] Thullner M. Kampara M. Richnow H. H. Harms H. Wick L. Y. (2008). Impact of bioavailability restrictions on microbially induced stable isotope fractionation. 1. Theoretical calculation. Environ. Sci. Technol. 42, 6544–6551. 10.1021/es702782c 18800528

[B100] Trudinge P. A. Chambers L. A. (1973). Reversibility of bacterial sulfate reduction and its relevance to isotope fractionation. Geochim. Cosmochim. Acta 37, 1775–1778 10.1016/0016-7037(73)90162-2

[B101] Tudge A. P. Thode H. G. (1950). Thermmodynamic properties of isotopic compounds of sulfur. Can. J. Res. Sec. B Chem. Sci. 28, 567–578 10.1139/cjr50b-069

[B102] Urey H. C. (1947). The thermodynamic properties of isotopic substances. J. Chem. Soc. 562–581. 10.1039/jr9470000562 20249764

[B103] Vairavamurthy A. Manowitz B. Luther G. W. Jeon Y. (1993). Oxidation state of sulfur in thiosulfate and implications for anaerobic energy metabolism. Geochim. Cosmochim. Acta 57, 1619–1623 10.1016/0016-7037(93)90020-W

[B104] Vairavamurthy M. A. Orr W. L. Manowitz B. (1995). Geochemical transformations of sedimentary sulfur: an introduction, in Geochemical Transformations of Sedimentary Sulfur, eds Vairavamurthy M. A. Schoonen M. A. A. (Washington, DC: American Chemical Society), 1–14 10.1021/bk-1995-0612.ch001

[B105] Venceslau S. S. Lino R. R. Pereira I. A. C. (2010). The Qrc membrane complex, related to the alternative complex III, is a menaquinone reductase involved in sulfate respiration. J. Biol. Chem. 285, 22772–22781. 10.1074/jbc.M110.124305 20498375 PMC2906268

[B106] Venceslau S. S. Stockdreher Y. Dahl C. Pereira I. A. C. (2014). The “bacterial heterodisulfide” DsrC is a key protein in dissimilatory sulfur metabolism. Biochim. Biophys. Acta-Bioenerg. 1837, 1148–1164. 10.1016/j.bbabio.2014.03.007 24662917

[B107] Wankel S. Bradley A. S. Eldridge D. Johnston D. T. (2013). Experimental determination of the equilibrium isotope effect between water and sulfite: implications for kinetic isotope fractionation in the sulfate reduction network. Geochim. Cosmochim. Acta 125, 694–711 10.1016/j.gca.2013.08.039

[B108] Widdel F. Bak F. (1992). Gram-negative mesophilic sulfate reducing bacteria, in The Prokaryotes, 2nd Edn, eds Balows T. Dworkin Harder (New York, NY: Springer), 1–1107.

[B109] Young E. D. Galy A. Nagahara H. (2002). Kinetic and equilibrium mass-dependent isotope fractionation laws in nature and their geochemical and cosmochemical significance. Geochim. Cosmochim. Acta 66, 1095–1104 10.1016/S0016-7037(01)00832-8

